# RDmap: a map for exploring rare diseases

**DOI:** 10.1186/s13023-021-01741-4

**Published:** 2021-02-25

**Authors:** Jian Yang, Cong Dong, Huilong Duan, Qiang Shu, Haomin Li

**Affiliations:** 1 The Children’s Hospital, Zhejiang University School of Medicine, National Clinical Research Center for Child Health, Binsheng Road 3333#, Hangzhou, Zhejiang, 310052 China; 2 The College of Biomedical Engineering and Instrument Science, Zhejiang University, Zhejiang, China

**Keywords:** Rare disease, Phenotype, Pathogenetic gene, Disease map, Clinical decision support

## Abstract

**Background:**

The complexity of the phenotypic characteristics and molecular bases of many rare human genetic diseases makes the diagnosis of such diseases a challenge for clinicians. A map for visualizing, locating and navigating rare diseases based on similarity will help clinicians and researchers understand and easily explore these diseases.

**Methods:**

A distance matrix of rare diseases included in Orphanet was measured by calculating the quantitative distance among phenotypes and pathogenic genes based on Human Phenotype Ontology (HPO) and Gene Ontology (GO), and each disease was mapped into Euclidean space. A rare disease map, enhanced by clustering classes and disease information, was developed based on ECharts.

**Results:**

A rare disease map called RDmap was published at http://rdmap.nbscn.org. Total 3287 rare diseases are included in the phenotype-based map, and 3789 rare genetic diseases are included in the gene-based map; 1718 overlapping diseases are connected between two maps. RDmap works similarly to the widely used Google Map service and supports zooming and panning. The phenotype similarity base disease location function performed better than traditional keyword searches in an in silico evaluation, and 20 published cases of rare diseases also demonstrated that RDmap can assist clinicians in seeking the rare disease diagnosis.

**Conclusion:**

RDmap is the first user-interactive map-style rare disease knowledgebase. It will help clinicians and researchers explore the increasingly complicated realm of rare genetic diseases.

## Background

Rare diseases commonly with a prevalence of less than 5 in 10,000 people [1], most of which are caused by underlying genetic factors, often manifest in infants or young children and affect the patients’ whole life. Although these conditions are rare, studies involving them have revealed important insights about normal physiology that, in turn, have provided a better understanding of common disorders, universal mechanisms, critical pathways, and therapies that are useful to treat more than one disease. However, correctly diagnosing rare genetic diseases is extremely complicated and remains a challenge in both developed and developing countries. According to a survey from EURORDIS [2], the interval from onset to diagnosis is 5 to 30 years for a quarter of patients with rare genetic diseases. During this period, the rate of first misdiagnosis is as high as 40%. If not corrected, these misdiagnoses would lead to a large number of invalid medical treatments or even unnecessary surgeries, seriously endangering the health of the patients and wasting medical resources at the same time. This highlights the need for accurate and timely diagnosis of rare diseases.

More than 7000 known rare diseases have been identified, and more than 100 novel disease-gene associations have been identified per year since the introduction of next-generation sequencing technologies [3]. The establishment of relationships between so many rare, complex and symptom-overlapping diseases from multiple levels such as phenotypic characteristics and molecular mechanisms is an important challenge of rare disease practice. Accumulating studies have found that genetic diseases that are caused by similar molecules [4–6] can be diagnosed by similar phenotypic characteristics [7, 8], and can ultimately be treated using similar drugs through corresponding targets [9–12]. Network-based medicine has emerged as a complementary approach for the identification of disease-causing genes, genetic mediators, and disruptions in the underlying cellular functions. Therefore, exploring the relationships among rare diseases can help to reveal the common attributes of similar rare genetic diseases. For example, the classification of rare diseases, phenotypic characteristics of diseases, and underlying genetic defects of genetic diseases can improve the probability of discovering potential pathogenic mechanisms and, most importantly, can help with the clinical diagnosis of rare genetic diseases and improve treatment plans.

In this study, we aimed to propose a method to construct two rare human disease maps based on the semantic similarities of both phenotypic characteristics and pathogenetic genes of rare diseases. Using advanced visualization technologies, the disease map can be used to reveal the complex relationships among different rare human genetic diseases and support the clinical diagnosis process.

## Results

**Fig. 1:**
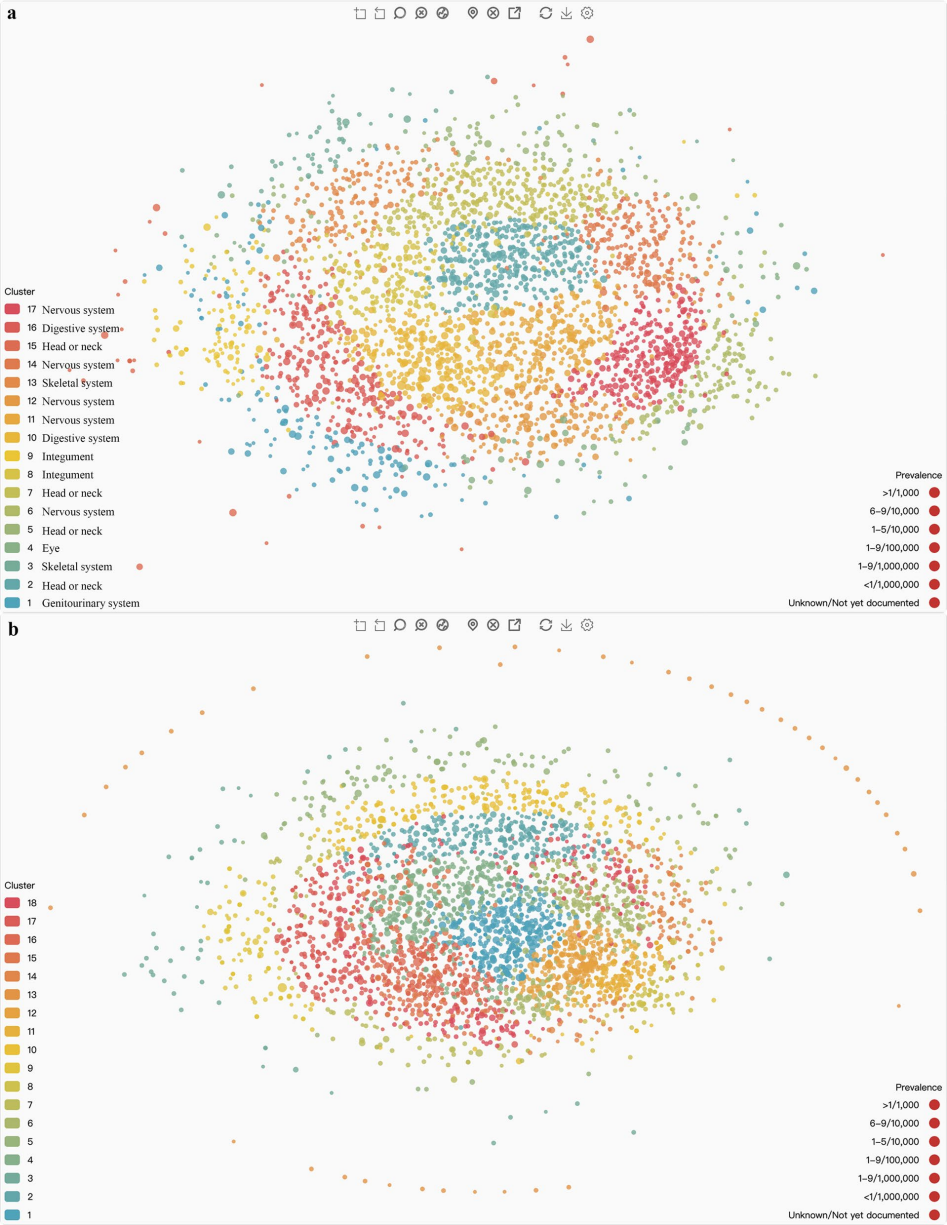
Rare disease maps and clusters (http://RDmap.nbscn.org). The locations reflect the distance among diseases, and the size of the points reflect the prevalence of rare diseases. **a** Rare disease map and clusters based on phenotype. The top affected systems were listed beside the cluster legends. **b** Rare disease map and clusters based on gene. More detail about the disease clusters and their relationships were available in the supplemental materials

In this study, 3287 diseases in Orphanet with a clinical phenotype and 3789 diseases with known pathogenic genes in Orphanet were plotted into Euclidean space, as shown in Fig. 1. In total, 17 phenotype-based disease clusters and 18 gene-based disease clusters were generated and highlighted by different colors. Detailed information on disease clustering is explained in the supplemental material.

**Fig. 2:**
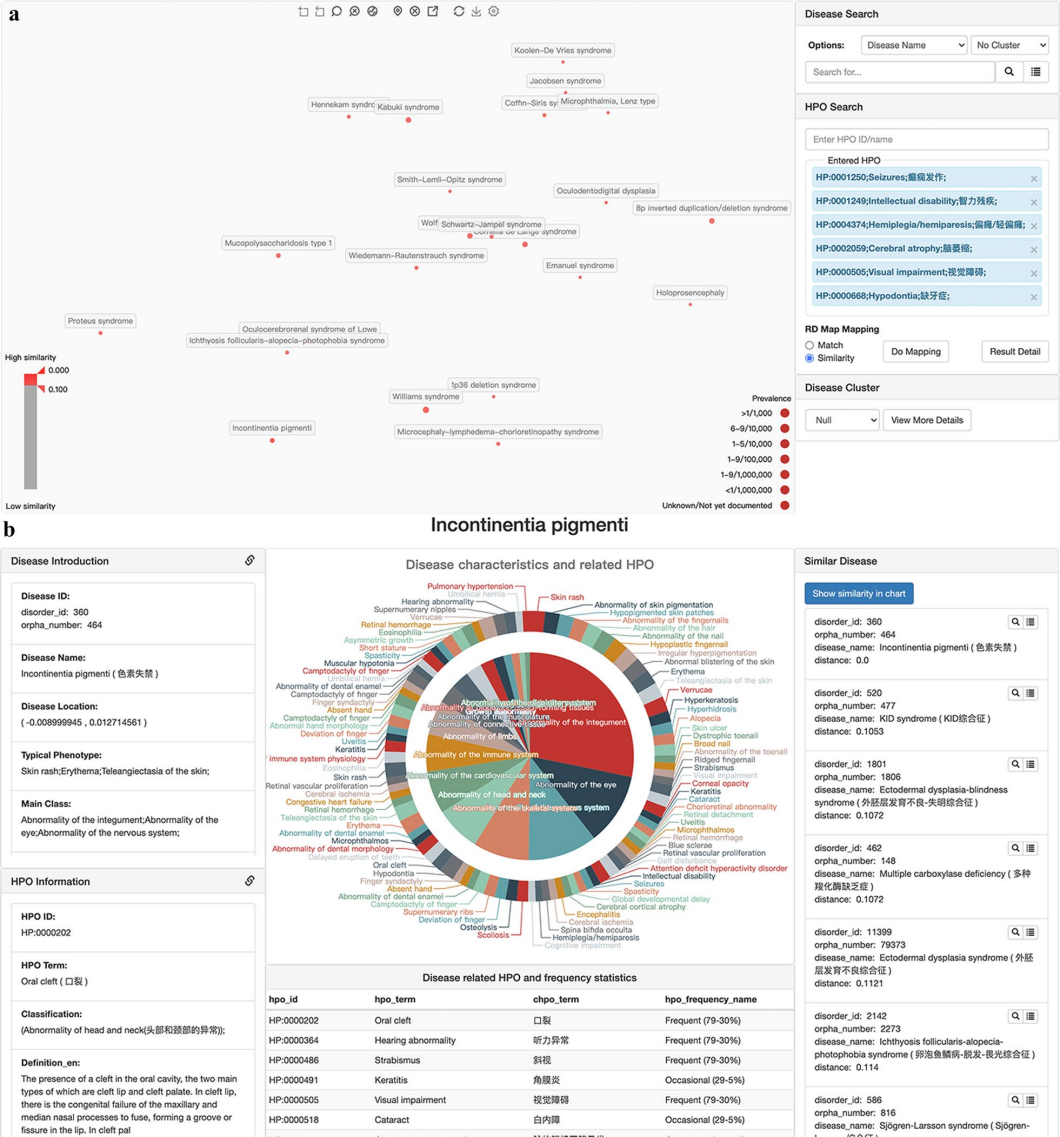
Rare disease map zooming, panning, location, filtering and disease detail information. **a** The RDmap locates similar diseases based on phenotype search. The slider in the left bottom corner can control the similarity filtering threshold by the user. The prevalence options switch at the bottom right can filter the results based on prevalence. **b** When a disease was selected on RDmap, its detail information will be shown like this

We published RDmap online (http://RDmap.nbscn.org) to help the user to explore rare disease relationships interactively. The map supports zooming and panning in the same manner as the widely used Google Maps service to find special diseases (Fig. 2). It also supports a feature-based exploration, such that one or more phenotypes will locate the most likely rare diseases on the map and filter by the similarity score (Fig. 2a). Detailed information about the disease is shown when the disease is confirmedly selected on the RDmap or clicking on the corresponding button (Fig. 2b). When a disease was selected on the RDmap, the user could jump between the phenotype map and gene map through a toolbar button. This will help users explore diseases of interest at different levels. An onboarding step-by-step user guide was developed on RDmap website to help users work on this novel tool.

**Fig. 3:**
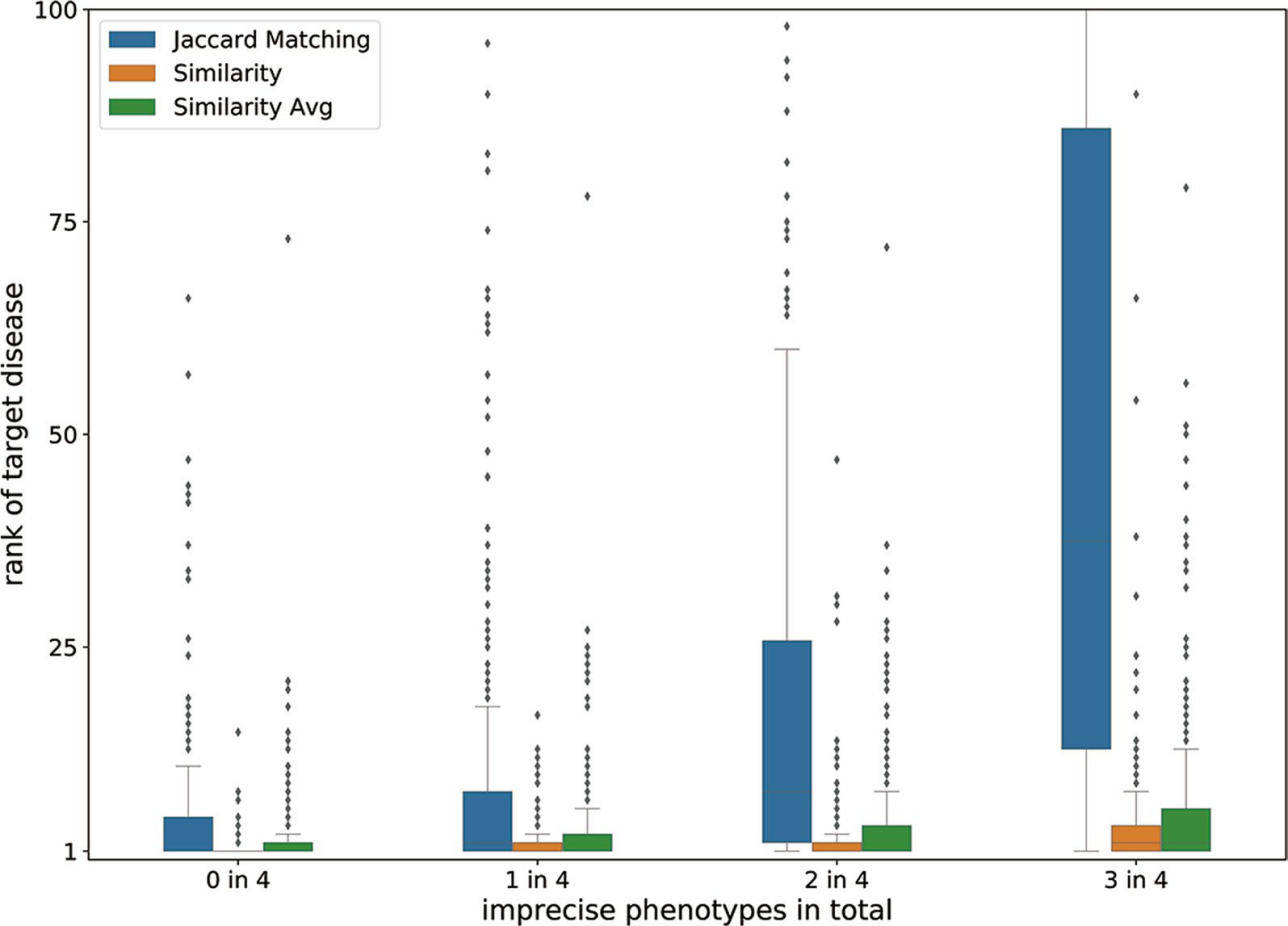
In silico test of RDMap. Performance of RDMap under conditions with different numbers of imprecision phenotypes for the search

In the in silico evaluation test, the performance of the Jaccard matching (direct phenotype term match) method decreases significantly as the number of imprecise phenotypes increases (Fig. 3). This finding also explains why it is very difficult to diagnose a rare genetic disease accurately in clinical practice using imprecise clinical phenotypes. The RDmap-proposed methods Similarity (one-way distance calculation) and Similarity-Avg (average of two-way distance calculation) both have an obvious advantage over the Jaccard matching method, particularly regarding imprecise phenotypes. We also noticed that the one-way distance algorithm (Similarity) is more stable in the disease recommendation than the Similarity-Avg in this scenario. This one-way distance algorithm was implemented in this published RDmap.

To further evaluate the performance of RDmap in clinical practice, a literature cases-based test was evaluated based on 20 published rare disease cases. The targeted diseases ranked in the similarity search results on RDmap are shown in Table 1 (the detailed information of each test case is shown in the supplemental material). RDmap worked pretty well in most cases with clear clinical phenotype descriptions. The average rank of targeted disease is 1.8 (median rank is 1, worse rank is 6) in 20 test cases. The similarity score (range from 0 to 1, the smaller the value, the more similar it is.) of the clinical phenotypes to targeted disease on RDmap is 0.031 ± 0.030 in these tests. If the user checks the detailed information of test case in the supplemental material, there are still diseases with identical similarity score in some test cases with top 1 rank. In clinical scenario, these candidate diseases will under consideration for the clinician. As all these similar diseases were highlighted on RDmap, a quick check of typical phenotypes and their frequency in these candidate diagnoses on RDmap will support clinicians in making a decision for real case.

**Table 1:** Evaluation of RDmap based on cases from publications

No	Author et al. Year	Disease (OMIM)	Phenotypes	Rank^1^	Sim. Score^2^
1	Al-Owain et al. 2010 [24]	Congenital disorder of glycosylation (OMIM 603147)	HP:0025356 Psychomotor retardation/Psychomotor HP:0001252 Muscular hypotonia HP:0001644 Dilated cardiomyopathy HP:0001250 Seizures HP:0000486 Strabismus HP:0006610 Wide intermammillary distance	6	0.0625
2	Böhm et al.2010 [25]	Centronuclear myopathies (OMIM 255200)	HP:0009073 Progressive proximal muscle weakness HP:0000297 Facial hypotonia HP:0000508 Ptosis HP:0000602 Ophthalmoplegia HP:0001315 Reduced tendon reflexes HP:0001256 Intellectual disability, mild	4	0.0486
3	Acién et al. 2010 [26]	Mayer-Rokitansky-Küster-Hauser syndrome (OMIM 277000)	HP:0002089 Pulmonary hypoplasia HP:0000122 Unilateral renal agenesis HP:0000151 Aplasia of the uterus HP:0008726 Hypoplasia of the vagina	4	0.0937
4	Mejia-Gaviria et al. 2010 [27]	Hereditary hypophosphatemic rickets with hypercalciuria (OMIM 241530)	HP:0002148 Hypophosphatemia HP:0002150 Hypercalciuria	1	0
5	Joy et al. 2007 [28]	Alström syndrome (OMIM 203800)	HP:0000662 Night blindness HP:0000618 Blindness HP:0012330 Pyelonephritis HP:0000822 Hypertension HP:0000819 Diabetes mellitus HP:0000510 Retinitis pigmentosa HP:0000518 Cataract	1	0.0535
6	Zhu et al. 2018 [29]	Cleidocranial dysplasia (OMIM 119600)	HP:0000684 Delayed eruption of teeth HP:0000164 Abnormality of the teeth HP:0000316 Hypertelorism HP:0011069 Increased number of teeth	1	0
7	Zamel et al. 2008 [30]	Abetalipoproteinemia (OMIM 200100)	HP:0002630 Fat malabsorption HP:0001251 Ataxia HP:0001324 Muscle weakness HP:0001315 Reduced tendon reflexes	4	0.0416
8	Vroegindeweij et al. 2020 [31]	Aceruloplasminemia (OMIM 604290)	HP:0001935 Microcytic anemia HP:0001260 Dysarthria HP:0001288 Gait disturbance HP:0000819 Diabetes mellitus HP:0001903 Anemia HP:0001300 Parkinsonism	1	0.0416
9	Zhou et al. 2018 [32]	Lymphangioleiomyomatosis (OMIM 606690)	HP:0100749 Chest pain HP:0002094 Dyspnea HP:0002107 Pneumothorax	1	0
10	Dias et al. 2016 [33]	Wolcott–Rallison syndrome (OMIM 226980)	HP:0006554 Acute hepatic failure HP:0001298 Encephalopathy HP:0000083 Renal insufficiency HP:0002654 Multiple epiphyseal dysplasia	1	0.0208
11	Valayannopoulos et al. 2010 [34]	Mucopolysaccharidosis type 6 (OMIM 253200)	HP:0000280 Coarse facial features HP:0000470 Short neck HP:0000158 Macroglossia HP:0002808 Kyphosis HP:0012471 Thick vermilion border	1	0.0083
12	Biesecker 2010 [35]	Greig cephalopolysyndactyly syndrome (OMIM 175700)	HP:0000256 Macrocephaly HP:0011304 Broad thumb HP:0001159 Syndactyly HP:0001162 Postaxial hand polydactyly HP:0005873 Polysyndactyly of hallux	1	0.016
13	Germain 2010 [36]	Fabry disease (OMIM 301500)	Angiokeratoma (HP:0001014)	1	0
14	Drera et al. 2009 [37]	Loeys-Dietz syndrome (OMIM 609192)	Camptodactyly of finger (HP:0100490) Ulnar deviation of the hand or fingers of the hand (HP:0001193) Bilateral talipes equinovarus (HP:0001776) Blue sclerae (HP:0000592) Microretrognathia (HP:0000308) High palate (HP:0000218) Bifid uvula (HP:0000193)	1	0.0628
15	Reibel et al. 2009 [38]	Hypophosphatasia (OMIM 146300)	Recurrent fractures (HP:0002757) Craniosynostosis (HP:0001363) Premature loss of teeth (HP:0006480)	1	0.0138
16	Sarfati et al. 2015 [39]	Kallmann syndrome (OMIM 308700)	Oligomenorrhea (HP:0000876) Breast hypoplasia (HP:0003187) Anosmia (HP:0000458) Hearing impairment (HP:0000365) Reduced number of teeth (HP:0009804)	1	0.0249
17	Weisfeld-Adams et al. 2013 [40]	Chédiak–Higashi syndrome (OMIM 214500)	Lower limb muscle weakness (HP:0007340) Dementia (HP:0000726) Ataxia (HP:0001251) Hypermetric saccades (HP:0007338) Bradykinesia (HP:0002067) Periodontitis (HP:0000704)	4	0.0972
18	Mowat et al. 2003 [41]	Mowat–Wilson syndrome (OMIM 235730)	Open mouth (HP:0000194) Abnormality of the eyebrow (HP:0000534) Frontal bossing (HP:0002007) Deeply set eye (HP:0000490) Wide nasal bridge (HP:0000431) Strabismus (HP:0000486)	1	0
19	Chrzanowska et al. 2012 [42]	Nijmegen breakage syndrome (OMIM 251260)	Microcephaly (HP:0000252) Sloping forehead (HP:0000340) Retrognathia (HP:0000278) Macrotia (HP:0000400) Bulbous nose (HP:0000414)	1	0.0116
20	Marshall et al. 2013 [43]	Wolfram syndrome (OMIM 222300)	Diabetes mellitus (HP:0000819) Optic atrophy (HP:0000648) Diabetes insipidus (HP:0000873) Hearing impairment (HP:0000365) Gastroesophageal reflux (HP:0002020)	1	0.0333

^1^Rank means the ranking of the target disease in the searching results on RDmap based on the phenotypes’ similarity scores. If there are identical similarity scores, the ranking is only calculated by the number of better scores

^2^Sim. Score means the similarity between the target disease and the input phenotypes. It is range from 0 to 1. The smaller the value, the more similar it is

## Discussion

**Fig. 4:**
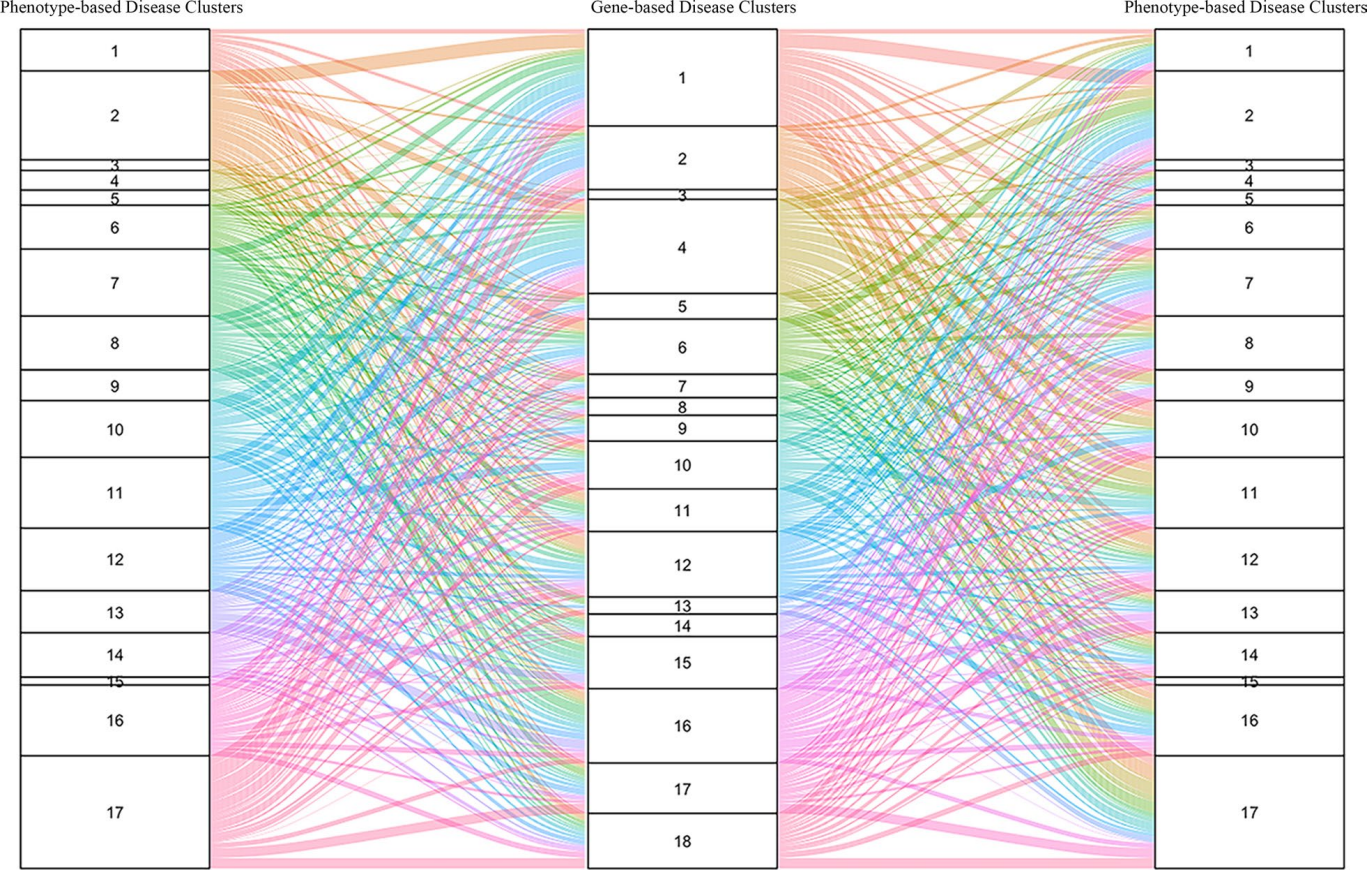
Alluvial diagram between 17 phenotype-based rare disease clusters and 18 gene-based rare disease clusters. The number shown in three columns represent the clusters N.O.; The width of the flow is the amount of diseases that overlapped in the connected phenotype-based disease cluster and gene-based disease cluster; The color of the flow was used to distinguish the source clusters

In this study, we constructed two maps of rare human genetic diseases based on phenotypic characteristics and genes and divided these genetic diseases into several disease clusters. Because diseases from the same cluster are related in phenotypic characteristics or gene functions, correlating clusters between two maps will be helpful to understand the physiological and pathological bases of related genetic diseases. Consistent with the results of Goh et al. [13], most of the diseases in the same phenotype-based cluster tend to have similar phenotypic characteristics. In total, 1718 diseases overlapped in the two maps, and the relationship between 17 phenotype-based clusters and 18 gene-based clusters is shown in an alluvial diagram in Fig. 4 and supplemental material. The complicated branches among these clusters further confirmed the complicated relationships among the pathogenic genes and phenotypes of rare genetic diseases. Diseases with similar phenotypes may be divided into different gene-based disease clusters. However, diseases from the same gene-based clusters also present diverse phenotypes. But, at the same time we also noticed mainstreams among different clusters. RDmap also provides a button to jump from disease selected in phenotype-based map to same disease in gene-based map and vice versa. Therefore, there are 1718 bridges between two maps. These findings will inspire researchers to evaluate the inner relationships among pathogenic genes and phenotypes.

In recent years, to reveal the similar relationships between different human genetic diseases, many studies have used various ways to construct a human genetic disease network. For example, Goh et al. extracted known disease-gene associations from the OMIM database and constructed the human disease network [13]. The core idea of their method is that two diseases are related if they share at least one common gene. Lee et al. constructed a human disease network based on cell metabolism, and the core idea of this method is that two diseases are related if the related mutant enzyme catalyzes the adjacent metabolism reaction [14]. Zhang et al. constructed a disease phenotype network using the similarity between phenotypes to obtain the gene function module [15]. Unlike these studies, RDmap shows a complicated disease relationship in a user-interactive map that we believe will be conducive to the discovery of potential relationships among pathogenic genes and phenotypic characteristics among many genetic diseases. The map-style visualization that reflects the distance of disease more intuitively will inspire investigators to understand the inner relationships among these diseases and their potential treatments and identify new pathogenic genes. In a traditional knowledge base, the entries are usually indexed by keywords, and users are required to use the exact term used in the knowledge base to query the knowledge. However, obtaining the exact phenotype features in a particular patient clinically and matching them with the standard phenotype terms used to annotate diseases in knowledgebases remain challenges [16]. Because thousands of genetic diseases are known, their clinical presentations often overlap in patients and are typically abridged with respect to classical descriptions. The incompleteness, heterogeneity, imprecision, and noise (the random co-occurrence phenotype) in phenotype description sometimes lead to missed diagnosis and even incorrect diagnoses. Based on two evaluation tests, this tool can help clinicians or genetic counselors accurately diagnose rare genetic diseases effectively, especially when the clinical phenotypes are incomplete, imprecise or noise.

This study has some limitations. First, the two disease maps still did not cover all rare genetic diseases. It is based on a history version of Orphanet in 2019 when this project started. Since then, there are about 69 new disease-gene associations and 782 new disease-phenotype associations updated in Orphanet. Second, when a novel disease is enrolled in the map, all the disease maps and disease clustering need to be recalculated and updated. However, we will update it annually based on feedback from the community.

## Conclusions

RDmap is the first user-interactive map-style rare disease knowledgebase. It also provides a disease search approach based on semantic similarity of phenotypes which will allow clinicians to identify potential rare disease with incompleteness, heterogeneity, imprecision, and even noise in phenotype description. Such a user-interactive network representations of rare diseases will help clinicians and researchers explore the increasingly complicated realm of rare genetic diseases.

## Methods

### Methods to measure the distance between phenotypes

Human Phenotype Ontology (HPO) [17] provides a standardized vocabulary that covers all the common abnormal phenotypes in humans and has been recognized as a useful annotation of the phenotypic abnormalities of rare genetic diseases. As with most modern ontologies, HPO is structured as a directed acyclic graph (DAG), whereby the nodes of the DAG, also called HPO terms, represent abnormal phenotypic terms in humans. Additionally, these phenotypic terms are linked to their parents through subclass (“is a”) relationships. In this study, we measured the distance between different phenotype terms based on the hierarchical structure of HPO. For any two HPO terms, the distance can be quantified by the shortest distance between the corresponding two nodes of the HPO DAG:

1 $$Dist_{p} \left( {p_{1} ,p_{2} } \right) = \frac{{\min (d_{1} + d_{2} )}}{{d_{\max } }}$$

where ${\text{d}}_{1}$ and ${\text{d}}_{2}$ represent the distances between two child nodes and their common parent nodes in the HPO DAG, respectively. Additionally, $d_{max}$ represents the maximum distance between nodes in the HPO DAG.

### Method to measure the distance between genes

The Gene Ontology (GO) knowledgebase is the world’s largest source of information on the functions of genes [18]. Similar to the above process, GO can be used to compute the distance between genes. GO describes genes from three different aspects: *molecular function*, *biological process* and *cell component*. Thus, the distance between any two genes from GO can be defined as the mean value of the shortest distance between gene nodes of the GO DAG from these three aspects:

2 $$Dist_{g} \left( {g_{1} ,g_{2} } \right) = \frac{{Dist_{cc} + Dist_{mf} + Dist_{bp} }}{3}$$

where $Dist_{cc}$, $Dist_{mf}$ and $Dist_{bp}$ represent the distance between two genes calculated by Formula 1 from three different aspects.

### Constructing the rare disease map based on Orphanet

Orphanet [19] was established in France in 1997 at the advent of the internet to gather scarce knowledge on rare diseases to improve the diagnosis, care and treatment of patients with rare diseases. Currently, Orphanet has become the reference source of information on rare diseases. In this study, 3287 diseases with a known clinical phenotype and 3789 diseases with known pathogenic genes, including 1718 overlapping diseases, were used to construct the rare disease map.

Because many rare diseases in Orphanet are annotated using HPO terms and frequency, each of these diseases can be represented by a set of phenotypes with weight. The phenotypic distance between disease ${d}_{1}$ and disease ${d}_{2}$ can be measured by Formula 3:

3 $$Dist_{dp} \left( {d_{1} ,d_{2} } \right) = \frac{1}{2}\left( {\frac{{\mathop \sum \nolimits_{i = 1}^{m} \mathop {\min }\limits_{1 \le j \le n} (Dist_{p} \left( {p_{i} ,p_{j} } \right)){*}\left( {w_{i} {*}w_{j} } \right)}}{m} + \frac{{\mathop \sum \nolimits_{i = 1}^{n} \mathop {\min }\limits_{1 \le j \le m} \left( {Dist_{p} \left( {p_{i} ,p_{j} } \right)} \right){*}\left( {w_{i} {*}w_{j} } \right)}}{n}} \right)$$

where ${\text{m}}$ and ${\text{n}}$ represent the number of phenotypes contained in disease $d_{1}$ and $d_{2}$, respectively, and $Dist\left( {p_{i} ,p_{j} } \right)$ represents the distance between two phenotypes $p_{i}$ and $p_{j}$ as shown in Formula 1, and $w_{i}$ and $w_{j}$ are the frequencies of two phenotypes $p_{i}$ and $p_{j}$ in $d_{1}$ and $d_{2}$, respectively.

Similarly, we extracted disease gene relationships from the Orphanet knowledgebase. The genetic distance between diseases can then be transformed into the distance between genes:

4 $$Dist_{dg} \left( {d_{1} ,d_{2} } \right) = \frac{1}{2}\left( {\frac{{\mathop \sum \nolimits_{i = 1}^{m} \mathop {\min }\limits_{1 \le j \le n} (Dist_{g} \left( {g_{i} ,g_{j} } \right))}}{m} + \frac{{\mathop \sum \nolimits_{i = 1}^{n} \mathop {\min }\limits_{1 \le j \le m} \left( {Dist_{g} \left( {g_{i} ,g_{j} } \right)} \right)}}{n}} \right)$$

where ${\text{m}}$ and ${\text{n}}$ represent the number of genes identified as pathogenic genes in diseases $d_{1}$ and $d_{2}$, respectively, and $Dist_{g} \left( {g_{i} ,g_{j} } \right)$ represents the distance between two genes $g_{i}$ and $g_{j}$, as shown in Formula 2.

By calculating these distances among all rare diseases from Orphanet, we generated two distance matrices with the sizes of $3287 \times 3287$ and $3789 \times 3789$ for phenotype and gene, respectively. We used multidimensional scaling [20] (*cmdscale* from the package *stats* in R [21]) to convert the distance matrix into 2D points, which can be visualized as a map.

To further explore the internal relationship between phenotypes and genes of rare genetic diseases, we divided the rare disease map into several disease clusters using the k-means clustering method. To determine the optimal k for disease clustering, a bootstrap approach implemented in the *clusterboot* function from the *fpc* package [22] in R was used.

Based on above mentioned data collection and processing, we developed a web-based interactive rare disease map based on ECharts [23] using Node.js. The similarity-based search engine was developed using Python. All other data processing were under R [21].

### Methods to evaluate the RDmap

To evaluate the RDmap in clinical diagnosis, we designed two evaluation tests. One is in silico test and the other is a literature case-based test.

In the in silico evaluation test, 1000 rare genetic diseases from the Orphanet database are taken as the target diseases. Then, each disease is represented as a set of four characteristic phenotypes with the highest frequency of the disease. In this in silico test, the adjacent node or parent node of the phenotype in the HPO DAG is defined as the imprecise phenotype of the target phenotype. We compared the semantic similarity based RDmap searching and the direct simple term matching based searching used in most of knowledge base on different precision level. The targeted disease ranked in the recommended disease list was used to evaluate the performance of RDmap.

In the literature case-based test, we collected 20 rare disease cases reported by the Orphanet Journal of Rare Diseases as test cases. These case reports were identified by search “case report” on the journal web site. The case presentations from the publications were manually converted to HPO terms by one of the authors. The targeted disease ranked in the recommended disease list by RDmap was used to evaluate the performance of RDmap. If there are identical similarity scores for several different diseases, the ranking is only calculated based on the number of diseases with better scores.

## Supplementary Information

**Additional file 1:** Supplemental Material.

## Data Availability

All data generated or analyzed during this study are published online or included in this published article and its supplementary files.

## Abbreviations

EURORDIS: Rare Diseases Europe
HPO: Human Phenotype Ontology
DAG: Directed acyclic graph
GO: Gene Ontology
OMIM: Online Mendelian Inheritance in Man
