# Supplementary material for: RDmap: a map for exploring rare diseases
Source: Orphanet J Rare Dis. 2021 Feb 25;16:101. doi: 10.1186/s13023-021-01741-4 (PMC7905868; doi:10.1186/s13023-021-01741-4)
Supplement: Supplementary file 1 — Additional file 1: Supplemental Material. [file 13023_2021_1741_MOESM1_ESM.pdf]

RDmap: A Map for Exploring Rare Diseases

Jian Yang, BS[#1,2]; Cong Dong, MS[#1,2]; Huilong Duan, PhD[2]; Qiang Shu, MD[1]; Haomin Li, PhD[1*]

Supplemental Material

1. Optimization of k for k-means clustering

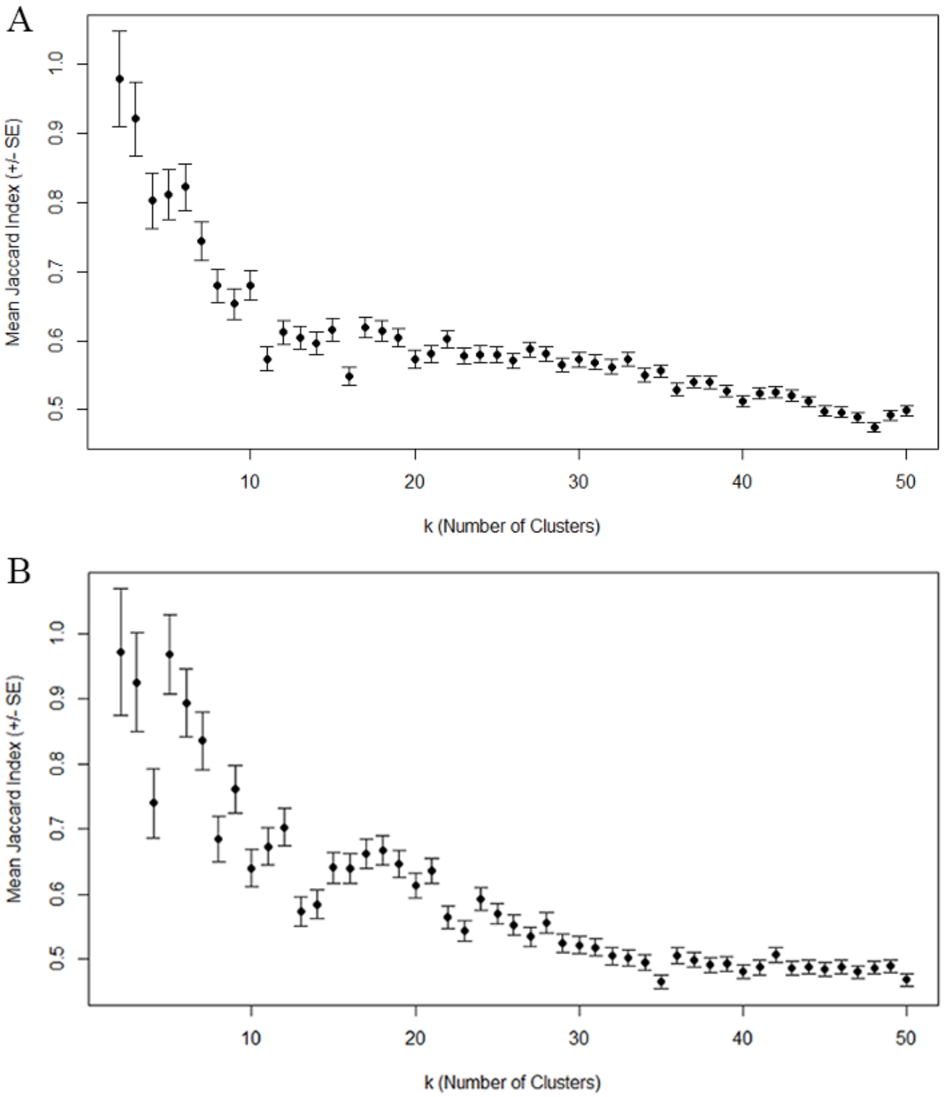

Figure S1. Results of disease clustering by using k-mean clustering algorithm. A. the disease clustering based on phenotype. B. the disease clustering based on gene

Using the *clusterboot* function (*fpc* package in R version 3.4.0) to clustering the distance matrix generated based on the methods described in main text with 100 repeats for each k from 2 to 50, the Jaccard index were shown in the figure S1 to evaluate the stable and separation degree among clusters. The larger the cluster separation degree between clusters,

the smaller the cluster cohesion, indicating the better the clustering effect. We also need to balance the clusters numbers (> 10) and the separation degree. Thus, based on the results in Fig S1, the disease map based on phenotype could be clustered into 17 clusters and the disease map based on gene could be clustered into 18 disease clusters.

2. Disease clusters

2.1 phenotypic disease clusters

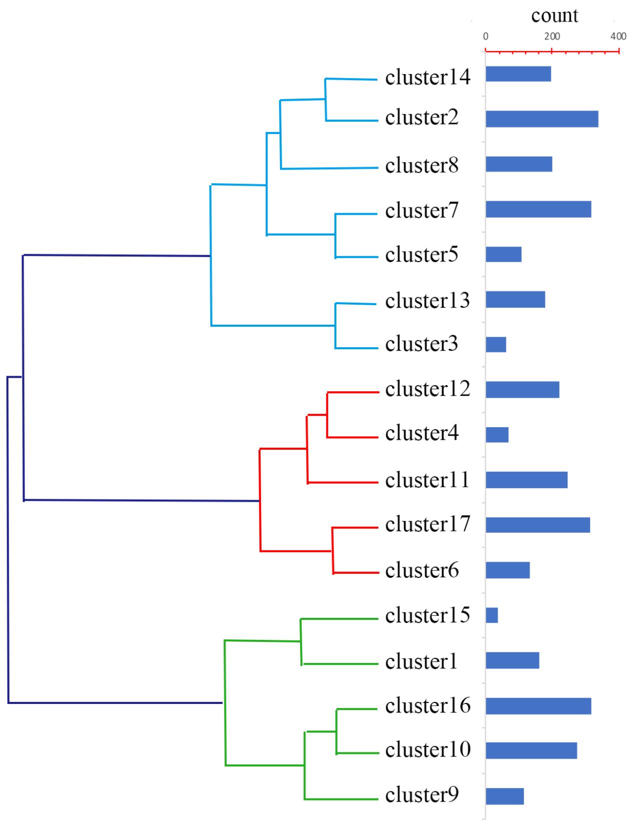

Figure S2. The phenotypic disease clusters

Table S1 The phenotypic rare disease clusters

Cluster #	count	Top 1 affected system	Top 5 HPO
Cluster 14	195	the nervous system	1. Intellectual disability (HP:0001249)<br>2. Seizures (HP:0001250)<br>3. Microcephaly (HP:0000252)<br>4. Global developmental delay (HP:0001263)<br>5. Short stature (HP:0004322)
Cluster 2	340	Head or neck	1. Short stature (HP:0004322)<br>2. Intellectual disability (HP:0001249)<br>3. Seizures (HP:0001250)<br>4. Microcephaly (HP:0000252)<br>5. Global developmental delay (HP:0001263)

Cluster 8	201	The integument	1. Short stature (HP:0004322)<br>2. Scoliosis (HP:0002650)<br>3. Intellectual disability (HP:0001249)<br>4. Abnormality of the teeth (HP:0000164)<br>5. Micrognathia (HP:0000347)
Cluster 7	317	Head or neck	1. Hypertelorism (HP:0000316)<br>2. Micrognathia (HP:0000347)<br>3. Short stature (HP:0004322)<br>4. Cleft palate (HP:0000175)<br>5. Intellectual disability (HP:0001249)
Cluster 5	106	Head or neck	1. Hypertelorism (HP:0000316)<br>2. Micrognathia (HP:0000347)<br>3. Cleft palate (HP:0000175)<br>4. Abnormality of dental morphology (HP:0006482)<br>5. Depressed nasal bridge (HP:0005280)
Cluster 13	180	The skeletal system	1. Short stature (HP:0004322)<br>2. Scoliosis (HP:0002650)<br>3. Micromelia (HP:0002983)<br>4. Joint stiffness (HP:0001387)<br>5. Abnormality of metaphyses (HP:0000944)
Cluster 3	61	The skeletal system	1. Finger syndactyly (HP:0006101)<br>2. Brachydactyly syndrome (HP:0001156)<br>3. Clinodactyly of the 5th finger (HP:0004209)<br>4. Camptodactyly of finger (HP:0100490)<br>5. Symphalangism affecting the phalanges of hand (HP:0009773)
Cluster 12	220	The nervous system	1. Seizures (HP:0001250)<br>2. Muscular hypotonia (HP:0001252)<br>3. Headache (HP:0002315)<br>4. Myopathy (HP:0003198)<br>5. Short stature (HP:0004322)
Cluster 4	70	Eye	1. Nystagmus (HP:0000639)<br>2. Visual impairment (HP:0000505)<br>3. Strabismus (HP:0000486)<br>4. Cataract (HP:0000518)<br>5. Photophobia (HP:0000613)
Cluster 11	246	The nervous system	1. Seizures (HP:0001250)<br>2. Intellectual disability (HP:0001249)<br>3. Ataxia (HP:0001251)<br>4. Muscular hypotonia (HP:0001252)

			5. Global developmental delay (HP:0001263)
Cluster 17	315	The nervous system	1. Seizures (HP:0001250)<br>2. Dysarthria (HP:0001260)<br>3. Ataxia (HP:0001251)<br>4. Hyperreflexia (HP:0001347)<br>5. Nystagmus (HP:0000639)
Cluster 6	131	The nervous system	1. Dysarthria (HP:0001260)<br>2. Hyperreflexia (HP:0001347)<br>3. Babinski sign (HP:0003487)<br>4. Ataxia (HP:0001251)<br>5. Difficulty walking (HP:0002355)
Cluster 15	36	Head or neck	1. Male infertility (HP:0003251)<br>2. Cleft palate (HP:0000175)<br>3. Gingival overgrowth (HP:0000212)<br>4. Sensorineural hearing impairment (HP:0000407)<br>5. Gingival fibromatosis (HP:0000169)
Cluster 1	160	Genitourinary system	1. Cryptorchidism (HP:0000028)<br>2. Ambiguous genitalia (HP:0000062)<br>3. Renal insufficiency (HP:0000083)<br>4. Male pseudohermarphroditism (HP:0000037)<br>5. Gynecomastia (HP:0000771)
Cluster 16	318	The digestive system	1. Hepatomegaly (HP:0002240)<br>2. Splenomegaly (HP:0001744)<br>3. Weight loss (HP:0001824)<br>4. Abdominal pain (HP:0002027)<br>5. Anemia (HP:0001903)
Cluster 10	275	The digestive system	1. Fatigue (HP:0012378)<br>2. Abdominal pain (HP:0002027)<br>3. Fever (HP:0001945)<br>4. Weight loss (HP:0001824)<br>5. Arthralgia (HP:0002829)
Cluster 9	116	The integument	1. Palmoplantar keratoderma (HP:0000982)<br>2. Abnormal blistering of the skin (HP:0008066)<br>3. Erythema (HP:0010783)<br>4. Papule (HP:0200034)<br>5. Pruritus (HP:0000989)

2.2 genetic disease clusters

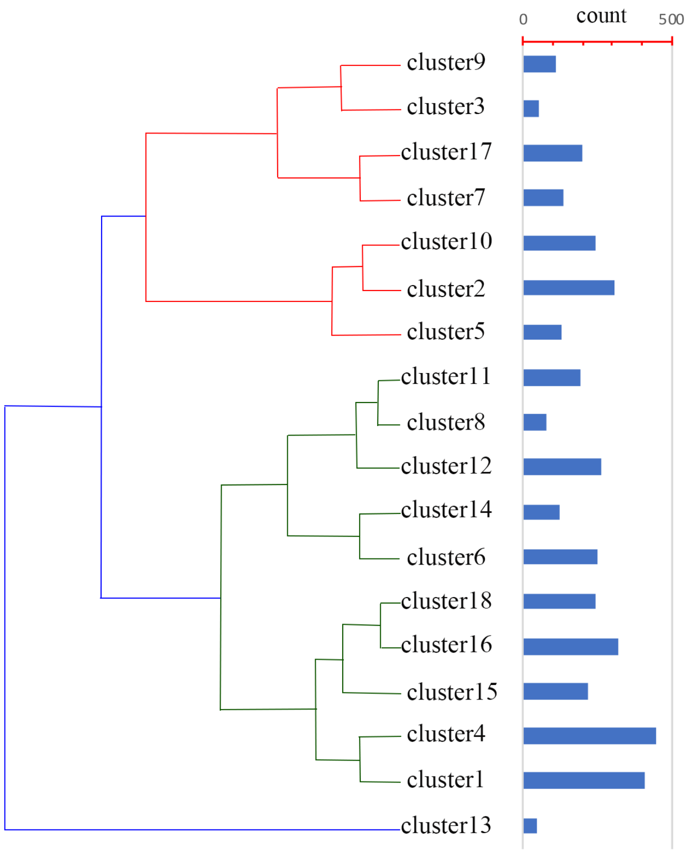

Figure S3. The genetic disease clusters

Cluster #	count	Top 5 Genes	Top 5 GO terms
Cluster 9	108	BEST1<br>SCN4A<br>SCN9A<br>SCN10A<br>SCN11A	1. establishment of localization (GO:0051234)<br>2. regulation of biology quality (GO:0065008)<br>3. regulation of biology process (GO:0050789)<br>4. system process (GO:0003008)<br>5. organic substance metabolic process (GO:0071704)
Cluster 3	55	CPT2<br>SLC35A2<br>GJC2<br>KANSL1<br>SCN11A	1. establishment of localization (GO:0051234)<br>2. regulation of biology process (GO:0050789)<br>3. organic substance metabolic process (GO:0071704)<br>4. primary metabolic process (GO:0044238)<br>5. cellular metabolic process (GO:0044237)
Cluster 17	199	RYR1<br>MEG3<br>DLK1<br>RTL1<br>GDAP1	1. regulation of biology process (GO:0050789)<br>2. establishment of localization (GO:0051234)<br>3. regulation of biology quality (GO:0065008)<br>4. response to chemical (GO:0065008)

			5. system process (GO:0003008)
Cluster 7	136	MYH7<br>LHCGR<br>IFNGR2<br>TBC1D24<br>KIF5A	1. regulation of biology process (GO:0050789)<br>2. signal transduction (GO:0007165)<br>3. cellular component organization (GO:0016043)<br>4. anatomical structure development (GO:0048856)<br>5. organic substance metabolic process (GO:0071704)
Cluster 10	245	IDH2<br>PAH<br>SI<br>HEXA<br>HADHA	1. organic substance metabolic process (GO:0071704)<br>2. cellular metabolic process (GO:0044237)<br>3. primary metabolic process (GO:0044238)<br>4. nitrogen compound metabolic process (GO:0006807)<br>5. small molecular metabolic process (GO:0044281)
Cluster 2	309	GBE1<br>PRPS1<br>FTL<br>UGT1A1<br>GBA	1. cellular metabolic process (GO:0044237)<br>2. organic substance metabolic process (GO:0071704)<br>3. primary metabolic process (GO:0044238)<br>4. nitrogen compound metabolic process (GO:0006807)<br>5. small molecular metabolic process (GO:0044281)
Cluster 5	131	PNPLA6<br>NAGA<br>CPA6<br>TSEN54<br>ST3GAL5	1. organic substance metabolic process (GO:0071704)<br>2. primary metabolic process (GO:0044238)<br>3. cellular metabolic process (GO:0044237)<br>4. nitrogen compound metabolic process (GO:0006807)<br>5. small molecular metabolic process (GO:0044281)
Cluster 11	194	ATRX<br>GATA1<br>HOXD13<br>PITX2<br>PITX1	1. regulation of biology process (GO:0050789)<br>2. anatomical structure development (GO:0048856)<br>3. cellular developmental process (GO:0048869)<br>4. anatomical structure morphogenesis (GO:0009653)

			5. organic substance metabolic process (GO:0071704)
Cluster 8	80	ARX<br>TBX4<br>NFIX<br>HOXA2<br>POU3F4	1. regulation of biology process (GO:0050789)<br>2. anatomical structure development (GO:0048856)<br>3. cellular developmental process (GO:0048869)<br>4. anatomical structure morphogenesis (GO:0009653)<br>5. cellular metabolic process (GO:0044237)
Cluster 12	262	PAX6<br>TP53<br>TP63<br>NKX2-5<br>GATA6	1. regulation of biology process (GO:0050789)<br>2. anatomical structure development (GO:0048856)<br>3. cellular developmental process (GO:0048869)<br>4. anatomical structure morphogenesis (GO:0009653)<br>5. signal transduction (GO:0007165)
Cluster 14	124	POLR3A<br>DICER1<br>RECQL4<br>SMARCAD1<br>MARS	1. organic substance metabolic process (GO:0071704)<br>2. cellular metabolic process (GO:0044237)<br>3. primary metabolic process (GO:0044238)<br>4. nitrogen compound metabolic process (GO:0006807)<br>5. regulation of biology process (GO:0050789)
Cluster 6	253	ERCC6<br>AIFM1<br>ERCC2<br>ERCC8<br>POLG	1. regulation of biology process (GO:0050789)<br>2. organic substance metabolic process (GO:0071704)<br>3. cellular metabolic process (GO:0044237)<br>4. primary metabolic process (GO:0044238)<br>5. nitrogen compound metabolic process (GO:0006807)
Cluster 18	244	PIK3CA<br>MPZ<br>HLA-DRB1<br>MFN2<br>PRKAR1A	1. regulation of biology process (GO:0050789)<br>2. signal transduction (GO:0007165)<br>3. cellular component organization (GO:0016043)<br>4. organic substance metabolic process

			(GO:0071704)<br>5. cellular metabolic process (GO:0044237)
Cluster 16	321	KIT<br>TRPV4<br>GDF5<br>FGFR3<br>LRP5	1. regulation of biology process (GO:0050789)<br>2. signal transduction (GO:0007165)<br>3. anatomical structure development (GO:0048856)<br>4. regulation of biology quality (GO:0065008)<br>5. cellular developmental process (GO:0048869)
Cluster 15	221	COL2A1<br>COL7A1<br>FBN1<br>ALPL<br>COL4A1	1. regulation of biology process (GO:0050789)<br>2. anatomical structure development (GO:0048856)<br>3. anatomical structure morphogenesis (GO:0009653)<br>4. cellular component organization (GO:0016043)<br>5. cellular developmental process (GO:0048869)
Cluster 4	451	HBB<br>GNAS<br>KRT1<br>KRT14<br>TTN	1. regulation of biology process (GO:0050789)<br>2. cellular component organization (GO:0016043)<br>3. establishment of localization (GO:0051234)<br>4. organic substance metabolic process (GO:0071704)<br>5. cellular metabolic process (GO:0044237)
Cluster 1	409	LMNA<br>PTEN<br>FUZ<br>VANGL1<br>MTHFD1	1. regulation of biology process (GO:0050789)<br>2. anatomical structure development (GO:0048856)<br>3. signal transduction (GO:0007165)<br>4. organic substance metabolic process (GO:0071704)<br>5. cellular metabolic process (GO:0044237)
Cluster 13	47	RNU4ATAC<br>MT-TL1<br>MT-TK<br>MT-TE<br>C12ORF65	1. nitrogen compound metabolic process (GO:0006807)<br>2. primary metabolic process (GO:0044238)<br>3. cellular metabolic process (GO:0044237)<br>4. organic substance metabolic process (GO:0071704)

			5. catabolic process (GO:0009056)

2.3 correlating clusters of phenotype and gene

The 1718 overlapping diseases between two rare disease maps were used for this correlating analysis. Expected the fig 4 shown in main text, the following 17 figures were used to show the 17 phenotypic disease clusters and their locations (x and y) on the gene-based disease map.

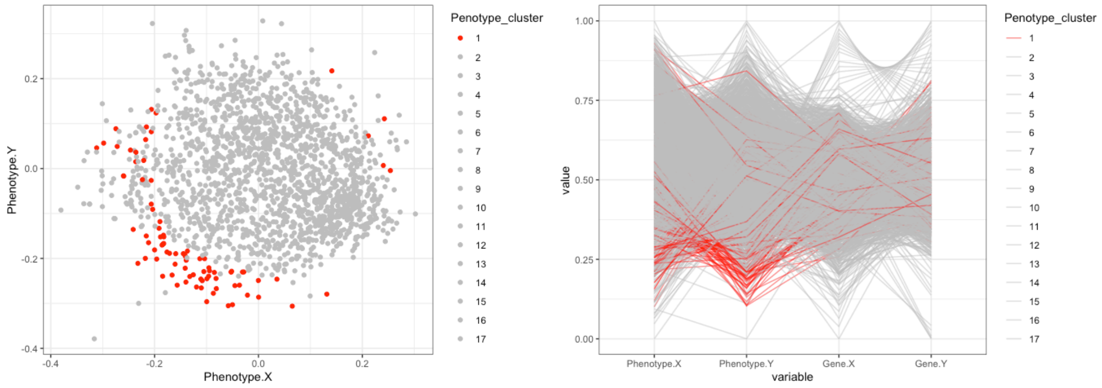

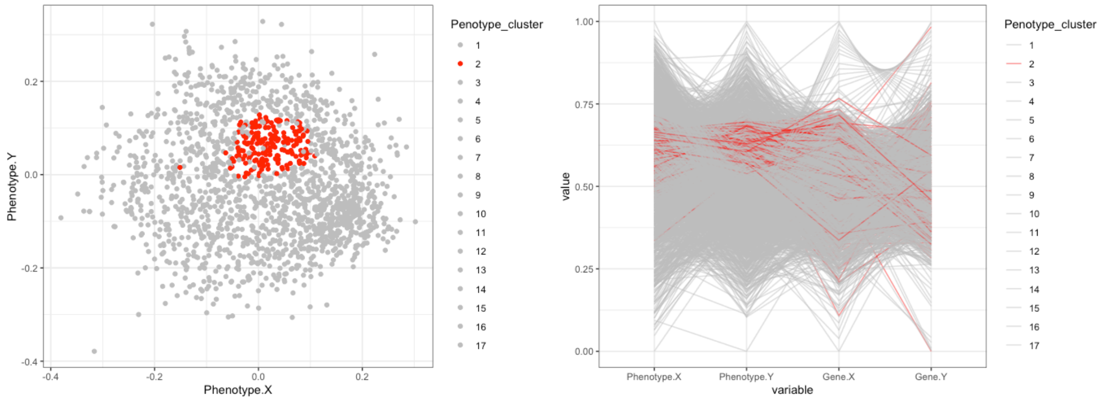

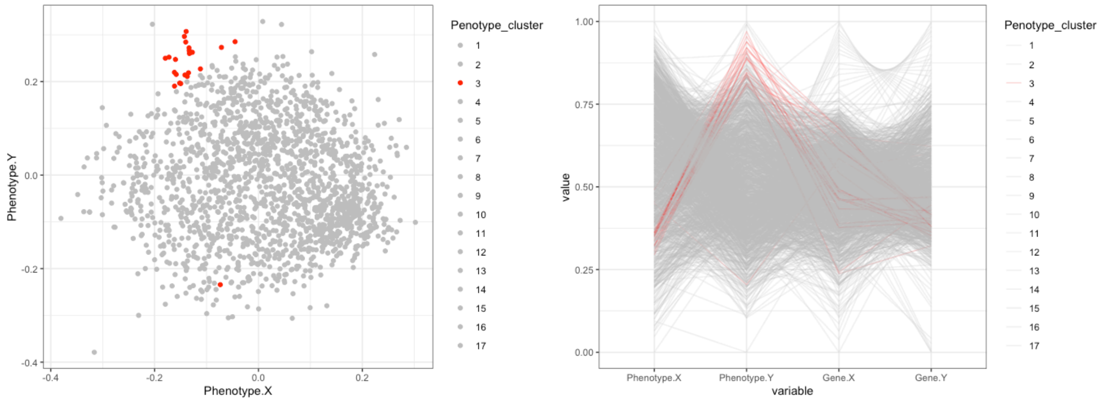

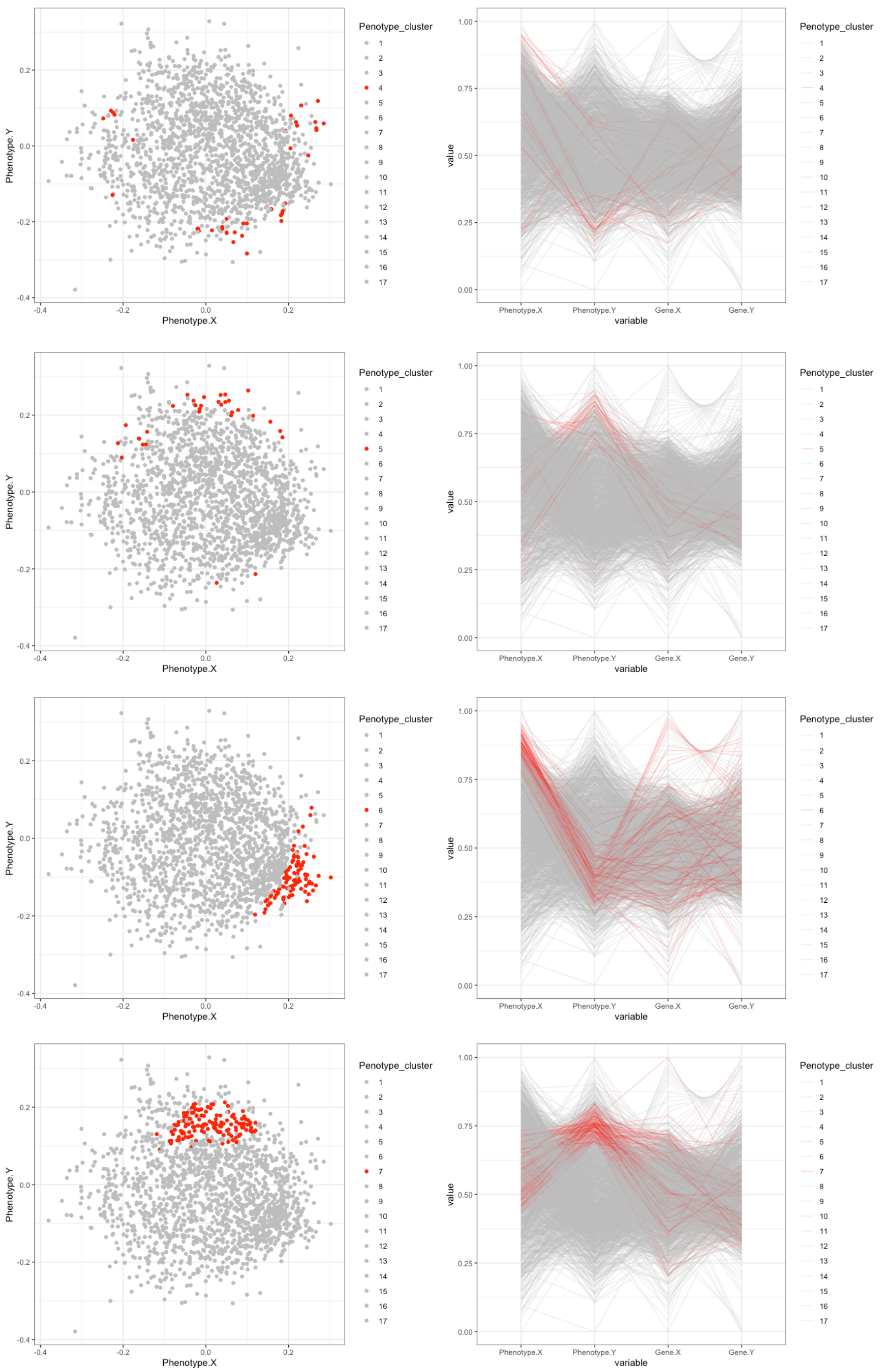
Penotype_cluster
1 2 3 4 5 6 7 8 9 10 11 12 13 14 15 16 17
Phenotype.Y
Phenotype.X
-0.4 -0.2 0.0 0.2
value
variable
Phenotype.X Phenotype.Y Gene.X Gene.Y
0.00 0.25 0.50 0.75 1.00

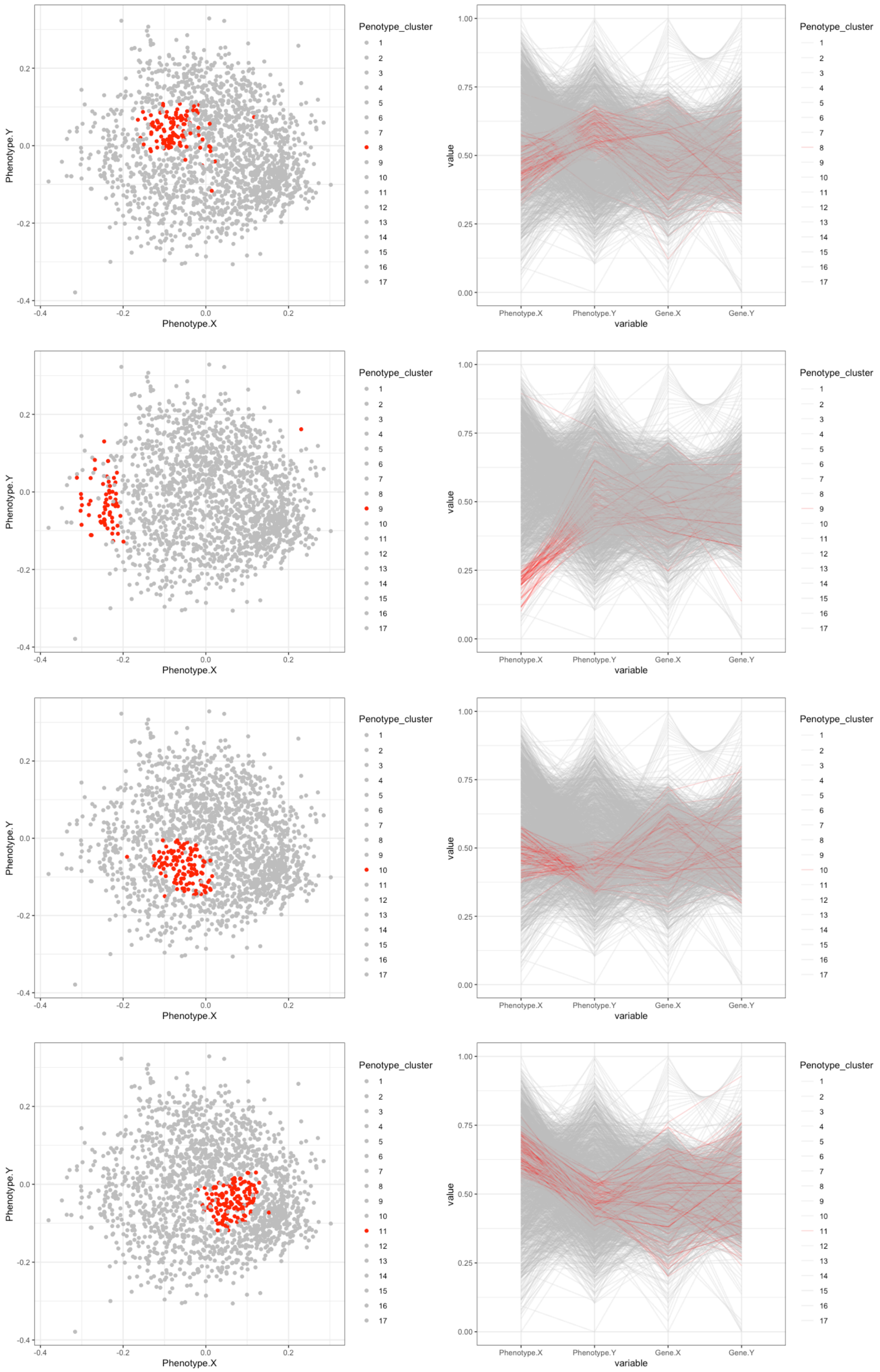
Penotype_cluster
1
2
3
4
5
6
7
8
9
10
11
12
13
14
15
16
17
Phenotype.Y
Phenotype.X
0.2
0.0
-0.2
-0.4
value
variable
1.00
0.75
0.50
0.25
0.00
Phenotype.X
Phenotype.Y
Gene.X
Gene.Y

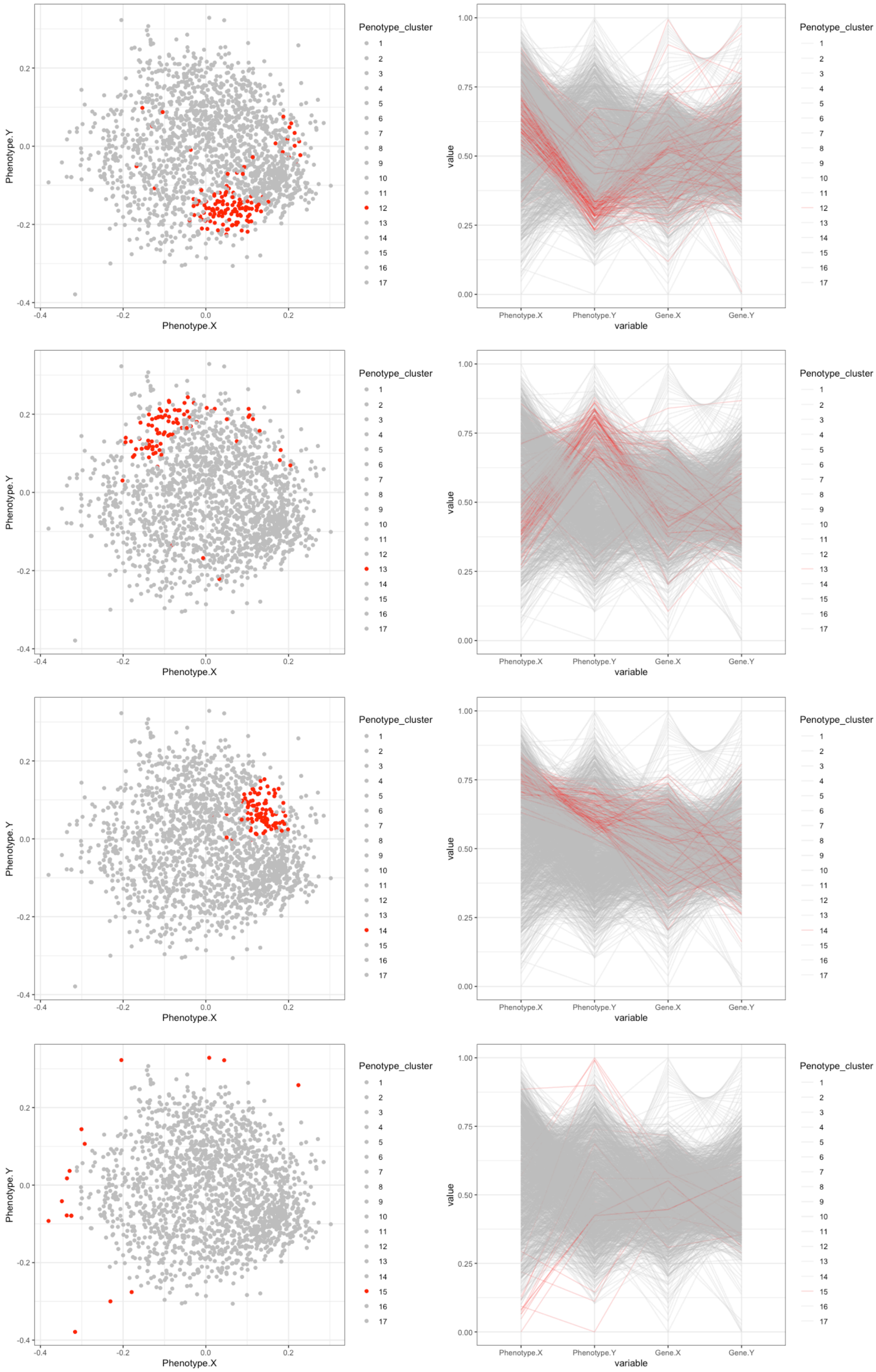

Penotype_cluster
1
2
3
4
5
6
7
8
9
10
11
12
13
14
15
16
17
Phenotype.X
Phenotype.Y
-0.4
-0.2
0.0
0.2
value
variable
Gene.X
Gene.Y
0.00
0.25
0.50
0.75
1.00

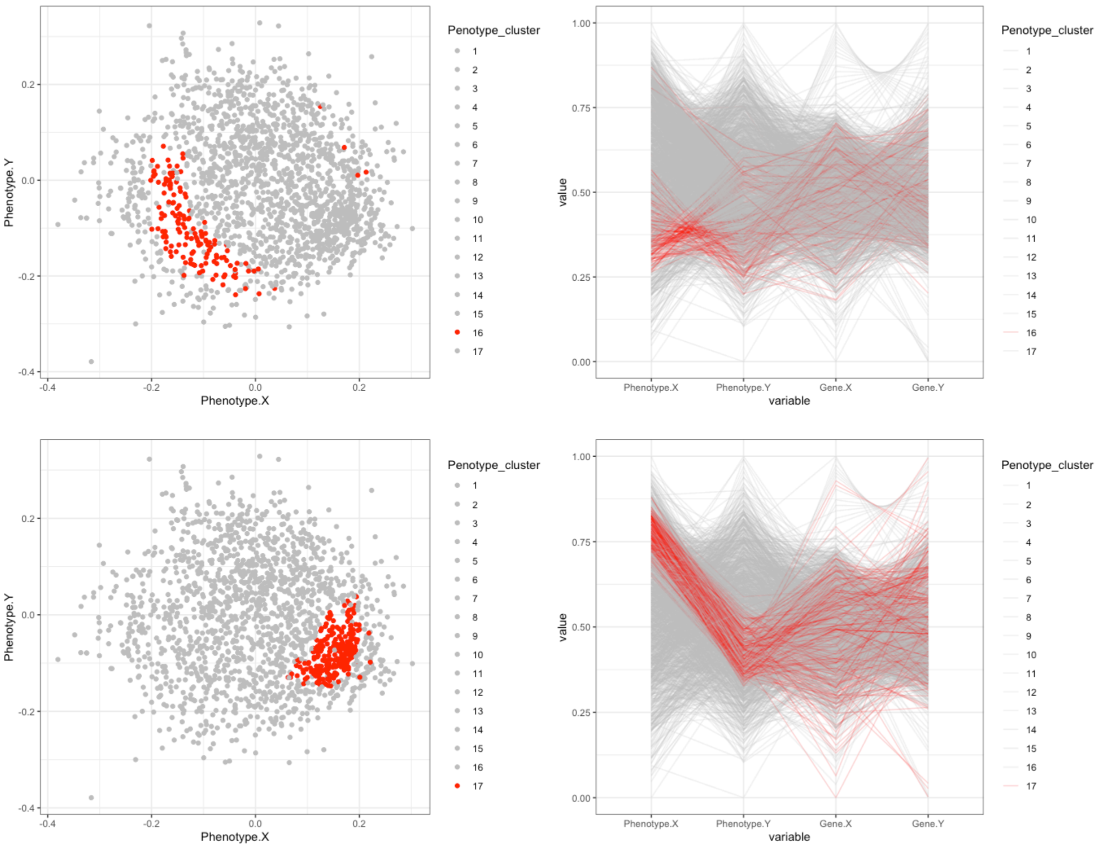

Figure S4. The phenotypic disease clusters and its distribution on the gene-based map

3. Detail of Test Cases

All rare disease cases were collected from the Orphanet Journal of Rare Disease using the "case report" as keywords for the query.
https://ojrd.biomedcentral.com/articles?query=case+report&volume=&searchType=&tab=keyword

As there are limited case reports, some cases that described in rare disease review were also included. Total 20 cases were collected.

Case 1:

Publication: Al-Owain, M., Mohamed, S., Kaya, N. et al. A novel mutation and first report of dilated cardiomyopathy in ALG6-CDG (CDG-Ic): a case report. Orphanet J Rare Dis 5, 7 (2010).

Case url: https://ojrd.biomedcentral.com/articles/10.1186/1750-1172-5-7

Diagnosis results: Congenital disorder of glycosylation

Case presentation:

A 9 year old Saudi boy (Fig. 1) was referred for evaluation of psychomotor retardation, hypotonia and dilated cardiomyopathy. He was born at 40 weeks gestation after a normal pregnancy and delivery with a birth weight of 4.6 kg. Hypotonia without feeding problems was noted in the neonatal period, bilateral alternating squint at two months of age, and hypokinesia at four months of age. At one year of age, he developed febrile seizures followed by afebrile partial epilepsy that responded well to carbamazepine. At three years of age, he presented with recurrent episodes of difficulty breathing and fatigability. Chest X-ray revealed cardiac enlargement with increased pulmonary vascularity. Echocardiography showed moderate dilatation and dysfunction of the left ventricle (LV). The end-systolic LV dimension was 3 (1.7-2.5 cm) corresponding to a Z-score of 4.3, while the end-diastolic LV dimension was 4.2 (2.9-3.9 cm) and the Z-score was 3.4. The ejection fraction and ejection fraction shortening were slightly subnormal at 56% and 27%, respectively. The interventricular septum thickness was normal. There was no mitral regurgitation and no pericardial effusion. These findings confirmed moderate cardiomyopathy of the dilated type. He was subsequently placed on captopril at a dose of 6.25 mg three times daily that was continued for five years. Captopril was just recently weaned off with stabilization of the cardiac function. At the age of 6 years, he was not able to sit unsupported, was nonverbal and is completely dependent on the family for care. On physical examination at the age of 7 years, the child was wheelchair bound with severe mental retardation and no speech. His head circumference and weight were on the 50 centile. His height was on the 25 centile. He had brachycephaly, bilateral esotropia, coarse hair with double hair whorl, low anterior hair line, broad nasal bridge, widely spaced eyes, prominent large ears, short philtrum, wide mouth with a thin upper lip, small teeth, widely spaced inverted nipples, bilateral cryptorchidism, reduced muscle bulk and tone with axial hypotonia. Deep tendon reflex were difficult to elicit. Finger joints were hyperextensible but knees and hips showed limited joint extension. Routine laboratory investigations showed normal urinalysis, normal complete blood count, blood glucose, thyroid and kidney function tests, serum amino acids, lactate, acylcarnitine profile, and urine organic acids.

Test result:

Entered HPO:

HP:0025356; HPO_term: Pschomotor retardation? /Psychomotor?; CHPO_term: 精神运动发育迟缓

HP:0001252; HPO_term: Muscular hypotonia; CHPO_term: 肌张力减退

HP:0001644; HPO_term: Dilated cardiomyopathy; CHPO_term: 扩张型心肌病

HP:0001250; HPO_term: Seizures; CHPO_term: 癫痫发作

HP:0000486; HPO_term: Strabismus; CHPO_term: 斜视

HP:0006610; HPO_term: Wide intermamillary distance; CHPO_term: 乳头间距宽

Mapping result:

disorder_id	orpha_number	disease_name	distance
3574	818	Smith-Lemli-Opitz syndrome（Smith-Lemli-Opitz综合征）	0.0416
1738	1606	1p36 deletion syndrome（1p36缺失综合征）	0.0486
19850	261112	Monosomy 9p（单体9p）	0.0486
19614	251071	8p23.1 microdeletion syndrome（8p23.1微缺失综合征）	0.0555
21498	314585	15q overgrowth syndrome（15q过度生长综合征）	0.0555
3553	137	Congenital disorder of glycosylation（先天性糖基化障碍）	0.0625
1056	10	48,XXYY syndrome（48，XXYY综合征）	0.0694

No.6

Figure C1-1 Mapping detail of Case1

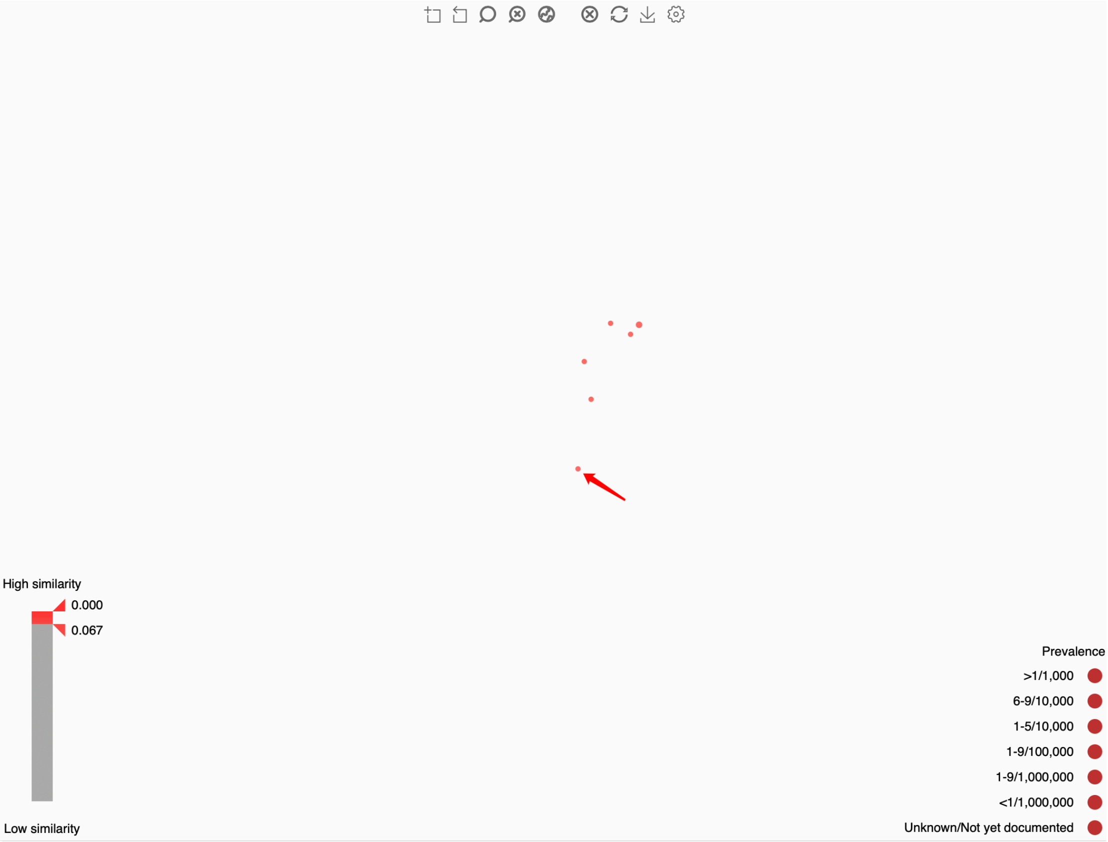

Figure C1-2 The target disease on RDmap of Case1

Case 2:

Publication: Böhm, J., Yiş, U., Ortaç, R. et al. Case report of intrafamilial variability in autosomal recessive centronuclear myopathy associated to a novel BIN1 stop mutation. Orphanet J Rare Dis 5, 35 (2010).

Case url: https://ojrd.biomedcentral.com/articles/10.1186/1750-1172-5-35

Diagnosis results: Centronuclear myopathies

Case presentation:

Patient 1 is a 13 year old girl belonging to a consanguineous family from Turkey without ancestral history of neuromuscular disorders. There were no complications during pregnancy, antenatal signs for muscle disorders as polyhydramnios and reduced fetal movements were not noted. Hypotonia was diagnosed at birth and motor development was delayed: head control was achieved at 6 months, walking at 18 months and running at 36 months. Muscle weakness was predominantly proximal, accompanied by mild facial weakness, ptosis and ophtalmoplegia/paresis. Tendinous reflexes were absent and she has no contractures. Although she has mild mental retardation (IQ 60), speech development was normal and she integrated the regular educational system.

Test result:

Entered HPO:

HP:0009073; HPO_term: Progressive proximal muscle weakness; CHPO_term: 进行性近端肌无力

HP:0000297; HPO_term: Facial hypotonia; CHPO_term: 面部肌张力低下

HP:0000508; HPO_term: Ptosis; CHPO_term: 上睑下垂

HP:0000602; HPO_term: Ophthalmoplegia; CHPO_term: 眼肌麻痹

HP:0001315; HPO_term: Reduced tendon reflexes; CHPO_term: 腱反射减低

HP:0001256; HPO_term: Intellectual disability, mild; CHPO_term: 轻度智力残疾

Mapping result:

disorder_id	orpha_number	disease_name	distance
13932	98915	Synaptic congenital myasthenic syndromes（突触先天性肌无力综合征）	0.0347
13931	98914	Presynaptic congenital myasthenic syndromes（突触前先天性肌无力综合征）	0.0416
8737	590	Congenital myasthenic syndrome（先天性肌无力综合征）	0.0416
63	550	MELAS（MELAS）	0.0486
17833	169186	Autosomal recessive centronuclear myopathy（常染色体隐性中心核肌病）	0.0486
8027	595	Centronuclear myopathy（中心核肌病）	0.0486

No.4

Figure C2-1 Mapping detail of Case2

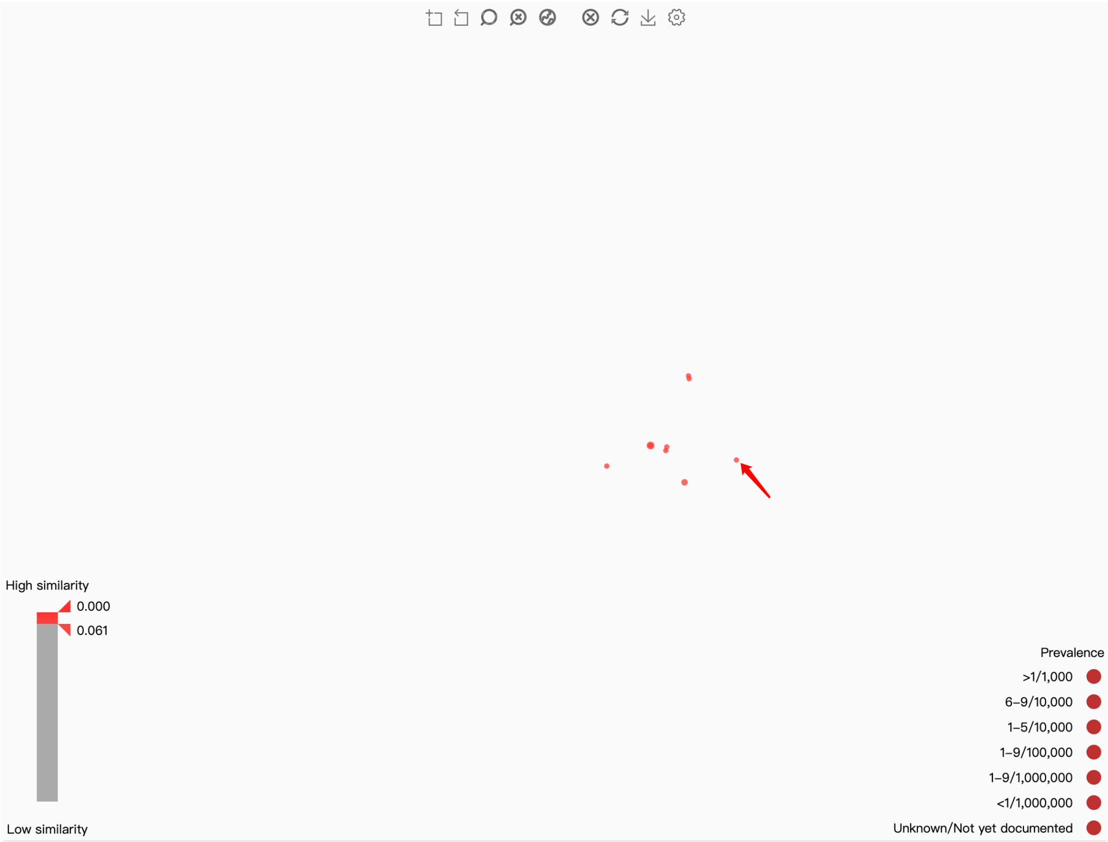

Figure C2-2 The target disease on RDmap of Case2

Case 3:

Publication: Acién, P., Galán, F., Manchón, I. et al. Hereditary renal adysplasia, pulmonary hypoplasia and Mayer-Rokitansky-Küster-Hauser (MRKH) syndrome: a case report. Orphanet J Rare Dis 5, 6 (2010).

Case url: https://ojrd.biomedcentral.com/articles/10.1186/1750-1172-5-6

Diagnosis results: Mayer-Rokitansky-Küster-Hauser syndrome

Case presentation:

A 17-year-old woman with primary amenorrhea was sent to us with a diagnosis of Rokitansky syndrome. The patient was born via normal delivery at a weight of 2400 g when her mother was 17. She was admitted to the hospital at an age of 8 days due to vomiting and moderate dystrophy; she was then diagnosed with primary right PH. She was later readmitted several times for pulmonary insufficiency. Two months later, a diagnosis of congenital right pulmonary hypoplasia with hypoplasia of the right lung artery was confirmed. In an ultrasound examination performed 7 months later, the right kidney was not observed. From more recent (at an age of 15 years) X-ray images taken of the pelvis in the Emergency Unit, the patient was also diagnosed with right hip dysplasia; this condition was initially defined as old secondary osteonecrosis of the right femoral head.The patient reported three years of genital and mammary development as well as cyclic pelvic pain for 4-5 days every month despite the primary amenorrhea. She weighed 42 kg and was 153 cm tall. The physical examination revealed normal external genital development and normal breasts. There was complete vaginal atresia. In the combined rectal examination, the pelvis was noted to be free. A transrectal ultrasound did not confirm the presence of a uterus. In this ultrasound examination, there was difficulty visualizing the ovaries; on the right side, however, vascular dilatations or multicystic embryonary remnants could be observed. The abdominal ultrasound did not show the presence of a right kidney.

Test result:

Entered HPO:

HP:0002089; HPO_term: Pulmonary hypoplasia; CHPO_term: 肺发育不良

HP:0000122; HPO_term: Unilateral renal agenesis; CHPO_term: 单侧肾缺如

HP:0000151; HPO_term: aplasia of the uterus; CHPO_term: 子宫发育不全

HP:0008726; HPO_term: Hypoplasia of the vagina; CHPO_term: 阴道发育不良

Mapping result:

disorder_id	orpha_number	disease_name	distance
2656	1848	Renal agenesis, bilateral（肾发育不全，双侧）	0.0729
1306	991	PAGOD syndrome（PAGOD综合征）	0.0729
603	887	VACTERL/VATER association（VACTERL / VATER关联）	0.0833
1814	1834	Axial mesodermal dysplasia spectrum（轴向中胚层发育不良谱）	0.0937
537	1505	Short rib-polydactyly syndrome（短肋多指综合征）	0.0937
287	289	Ellis Van Creveld syndrome（埃利斯·范·克雷维德综合征）	0.0937
2783	3109	Mayer-Rokitansky-Küster-Hauser syndrome（Mayer-Rokitansky-Kü	0.0937

No.4

Figure C3-1 Mapping detail of Case3

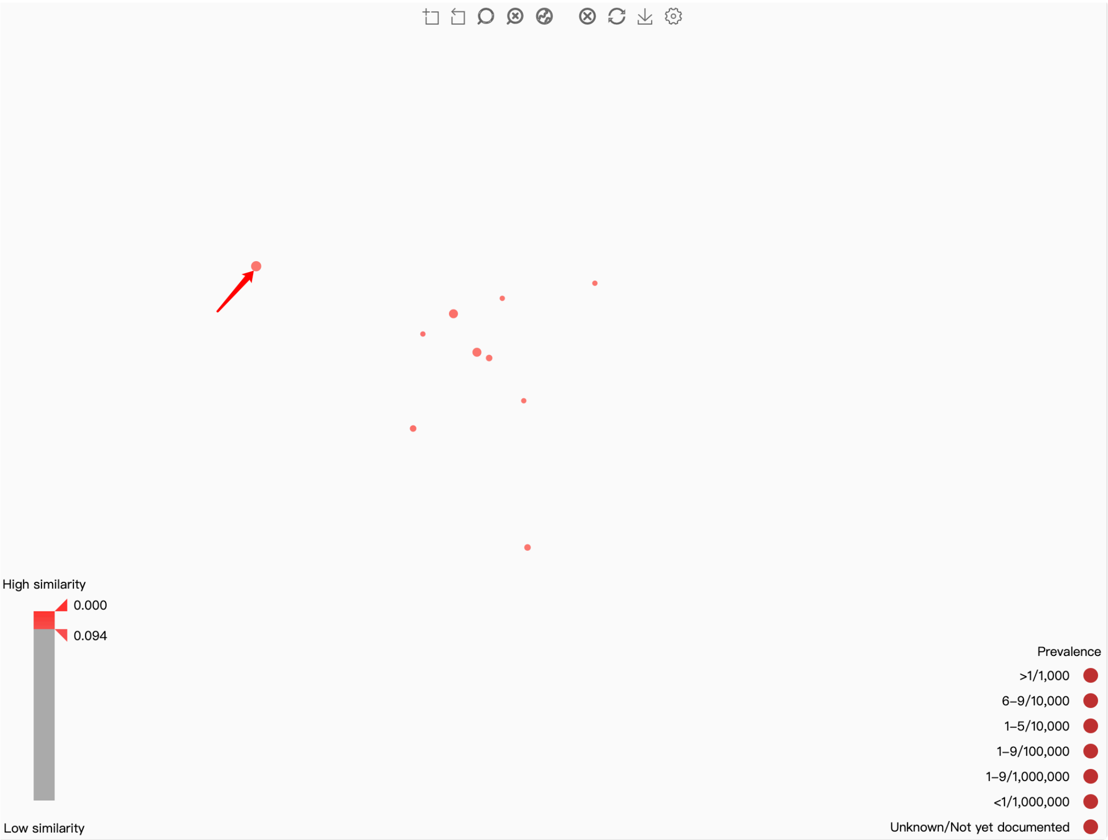

Figure C3-2 The target disease on RDmap of Case3

Case 4:

Publication: Mejia-Gaviria, N., Gil-Peña, H., Coto, E. et al. Genetic and clinical peculiarities in a new family with hereditary hypophosphatemic rickets with hypercalciuria: a case report. Orphanet J Rare Dis 5, 1 (2010).

Case url: https://ojrd.biomedcentral.com/articles/10.1186/1750-1172-5-1

Diagnosis results: Hereditary hypophosphatemic rickets with hypercalciuria

Case presentation:

A four member Spanish family is described in this report. Parents were not consanguineous and all members were asymptomatic. The daughter was referred to our outpatient clinic at the age of 11 years for evaluation of hyperphosphatasia and hypercalciuria. She had been suffering of intermittent abdominal pain for 3 years but denied bone pain or renal colic. At the first evaluation her height was 153.3 cm (50th percentile) and her weight 44.5 kg (50th percentile), normal blood pressure (116/60 mm Hg) and the only abnormal finding in the physical examination was a mild dorsal scoliosis. The initial serum biochemistry in our clinic confirmed the previous biochemical alterations and showed decreased tubular reabsorption of phosphate (TRP) and high circulating levels of 1,25 dihydroxyvitamin D. Acid-base status and serum urea, creatinine, albumin, lipid profile and liver-function tests were normal. Bilateral medullar nephrocalcinosis was found by renal ultrasonography. No radiological signs of rickets were present, bone mineral density was within normal reference values and bone age was according to chronological age. Oral phosphate supplementation was prescribed but only for a short period because of patient's bad compliance and lack of major disease-related manifestations. Renal ultrasound of the parents and brother were normal. After a 7.5 year follow up, the four family members continue asymptomatic and their initial laboratory alterations remain essentially unchanged.

Test result:

Entered HPO:

HP:0002148; HPO_term: Hypophosphatemia; CHPO_term: 低磷血症

HP:0002150; HPO_term: Hypercalciuria; CHPO_term: 高钙尿症

Mapping result:

disorder_id	orpha_number	disease_name	distance
14452	99879	Familial isolated hyperparathyroidism（家族性孤立性甲状旁腺功能亢进症）	0.0
8675	143	Parathyroid carcinoma（甲状旁腺癌）	0.0
14453	99880	Hyperparathyroidism-jaw tumor syndrome（甲状旁腺功能亢进-颌骨肿瘤综合征）	0.0
17137	157215	Hereditary hypophosphatemic rickets with hypercalciuria（遗传性低磷血症佝偻病伴高钙尿症）	0.0 No.1
123	534	Oculocerebrorenal syndrome of Lowe（劳氏眼脑肾综合征）	0.0

Figure C4-1 Mapping detail of Case4

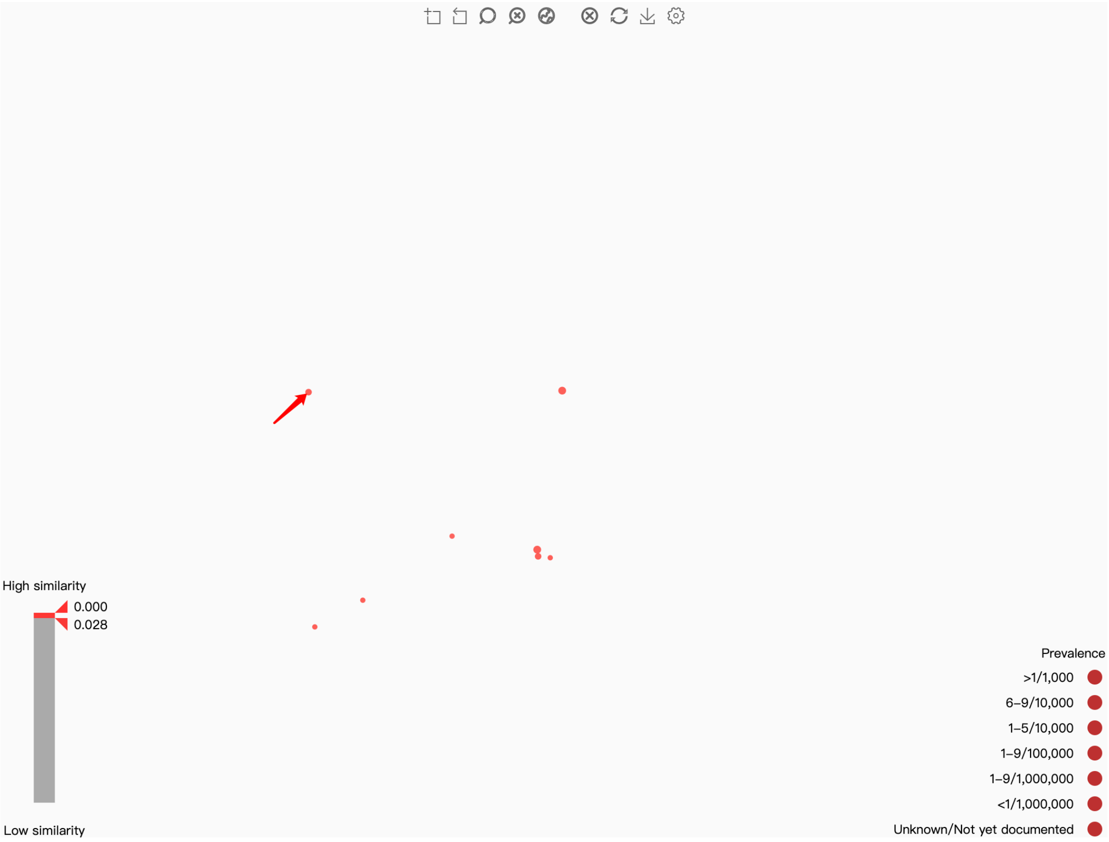

Figure C4-2 The target disease on RDmap of Case4

Case 5:

Publication: Joy, T., Cao, H., Black, G. et al. Alstrom syndrome (OMIM 203800): a case report and literature review. Orphanet J Rare Dis 2, 49 (2007).

Case url: https://ojrd.biomedcentral.com/articles/10.1186/1750-1172-2-49

Diagnosis results: Alstrom syndrome

Case presentation:

In 2002, the 27-year old proband was referred to the lipid clinic of a tertiary health care centre for evaluation of an elevated triglyceride (TG) level of 59.1 mmol/L. Her prior history included poor vision since birth, commencing with the development of night blindness, eventually resulting in legal blindness by the age of 17. She had undergone a left nephrectomy at the age of 24 for a perinephric abscess due to chronic pyelonephritis. Ultrasound evaluation revealed a normal-sized right kidney with evidence of cortical scarring. Hypertension and diabetes subsequently developed at the ages of 25 and 26 years, respectively. She experienced learning difficulties in school, but did not have sensorineural deafness. On physical examination, there was evidence of central obesity with her body mass index (BMI) being 34.9 kg/m2. Her blood pressure on antihypertensive treatment was 132/86 with a regular pulse of 80 beats per minute. There was no evidence of poly- or syndactyly suggestive of Bardet-Biedl syndrome. Hirsutism was present on the face, abdomen, and arms. Ophthalmologic examination was notable for retinitis pigmentosa and cataracts bilaterally.

Test result:

Entered HPO:

HP:0000662; HPO_term: Night blindness; CHPO_term: 夜盲症

HP:0000618; HPO_term: Blindness; CHPO_term: 失明

HP:0012330; HPO_term: Pyelonephritis; CHPO_term: 肾盂肾炎

HP:0000822; HPO_term: Hypertension; CHPO_term: 高血压

HP:0000819; HPO_term: Diabetes mellitus; CHPO_term: 糖尿病

HP:0000510; HPO_term: Retinitis pigmentosa; CHPO_term: 视网膜色素变性

HP:0000518; HPO_term: Cataract; CHPO_term: 白内障

Mapping result:

disorder_id	orpha_number	disease_name	distance	
63	550	MELAS（MELAS）	0.0535	No.1
1328	64	Alström syndrome（阿尔斯特伦症候群）	0.0535	
7037	225	Maternally-inherited diabetes and deafness（母亲遗传性糖尿病和耳聋）	0.0595	
185	636	Neurofibromatosis type 1（1型神经纤维瘤病）	0.0654	
99	892	Von Hippel-Lindau disease（冯·希佩尔·林道病）	0.0773	
145	904	Williams syndrome（威廉姆斯综合症）	0.0773	

Figure C5-1 Mapping detail of Case5

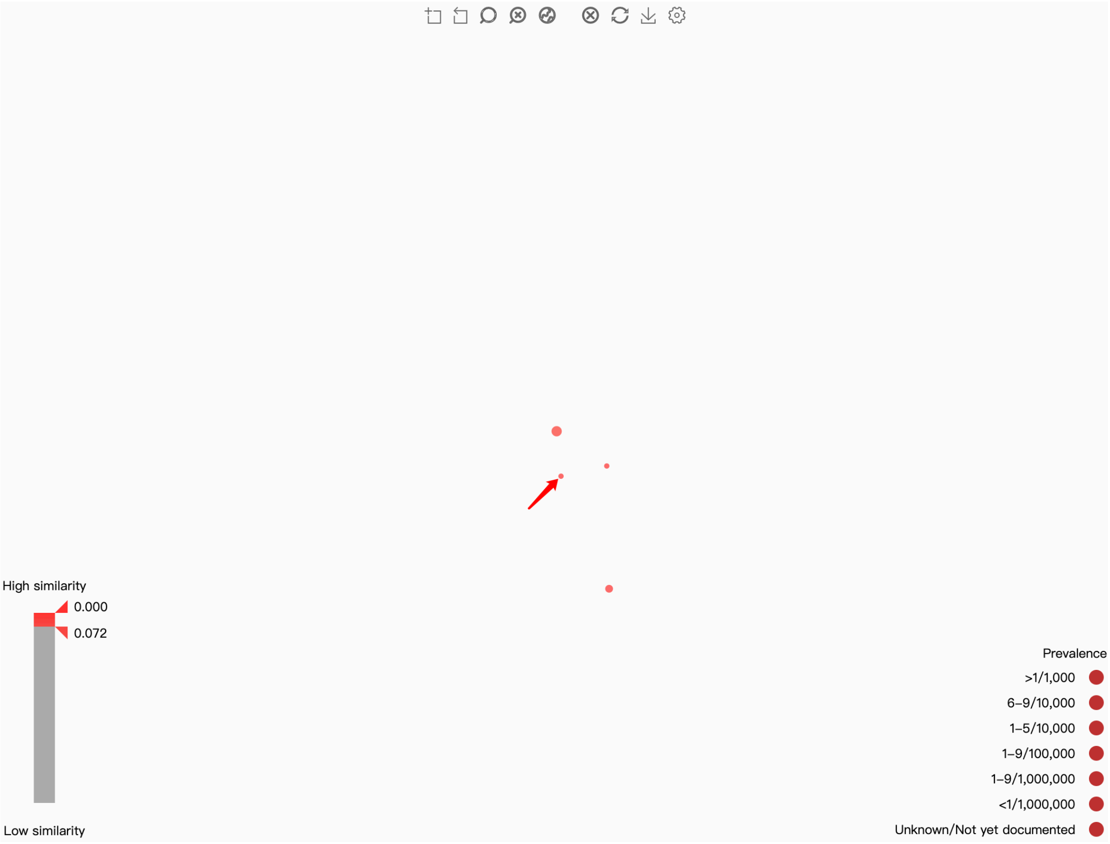

Figure C5-2 The target disease on RDmap of Case5

Case 6:

Publication: Zhu, Y., Zou, Y., Yu, Q. et al. Combined surgical-orthodontic treatment of patients with cleidocranial dysplasia: case report and review of the literature. Orphanet J Rare Dis 13, 217 (2018).

Case url: https://ojrd.biomedcentral.com/articles/10.1186/s13023-018-0959-3

Diagnosis results: Cleidocranial dysplasia

Case presentation:

A 16–year–old female came for an orthodontic consultation in March, 2008 with chief complains of crossbite and failure of eruption of permanent dentition.

Intraoral examination showed a mixed dentition with Class III malocclusion. The overjet was -3 mm, overbite was − 7 mm and the midlines were centered with no notable shift.

The panoramic radiograph revealed congenitally missing one lower incisor, the ectopic localization of permanent teeth, cysts formation involving the mandibular premolars, and the presence of 7 supernumerary teeth (1 in the maxilla and 6 in the mandible). The lateral radiograph confirmed a skeletal Class III malocclusion caused by mandibular hyperplasia and rotation with a horizontal growth (ANB = − 1°; Wits = − 0.3 mm; FMA = 20.2°).

This patient was diagnosed with cleidocranial dysplasia based on the presence of pathognomonic appearance, hypoplasia of clavicles, failure of permanent teeth eruption, and multiple supernumerary teeth.

Test result:

Entered HPO:

HP:0000684; HPO_term: Delayed eruption of teeth; CHPO_term: 牙齿萌出延迟

HP:0000164; HPO_term: Abnormality of the teeth; CHPO_term: 牙齿异常

HP:0000316; HPO_term: Hypertelorism; CHPO_term: 眼距过宽

HP:0011069; HPO_term: Increased number of teeth; CHPO_term: 牙齿数量增加

Mapping result:

disorder_id	orpha_number	disease_name	distance	No.1
443	1452	Cleidocranial dysplasia（颅骨发育不良）	0.0	
2036	2136	Hennekam syndrome（Hennekam综合征）	0.0104	
1674	1507	Autosomal recessive Robinow syndrome（常染色体隐性Robinow综合征）	0.0208	
472	235	Dubowitz syndrome（Dubowitz综合征）	0.0208	
394	915	Aarskog-Scott syndrome（Aarskog-Scott综合征）	0.0208	
3498	3474	CHIME syndrome（CHIME综合征）	0.0208	

Figure C6-1 Mapping detail of Case6

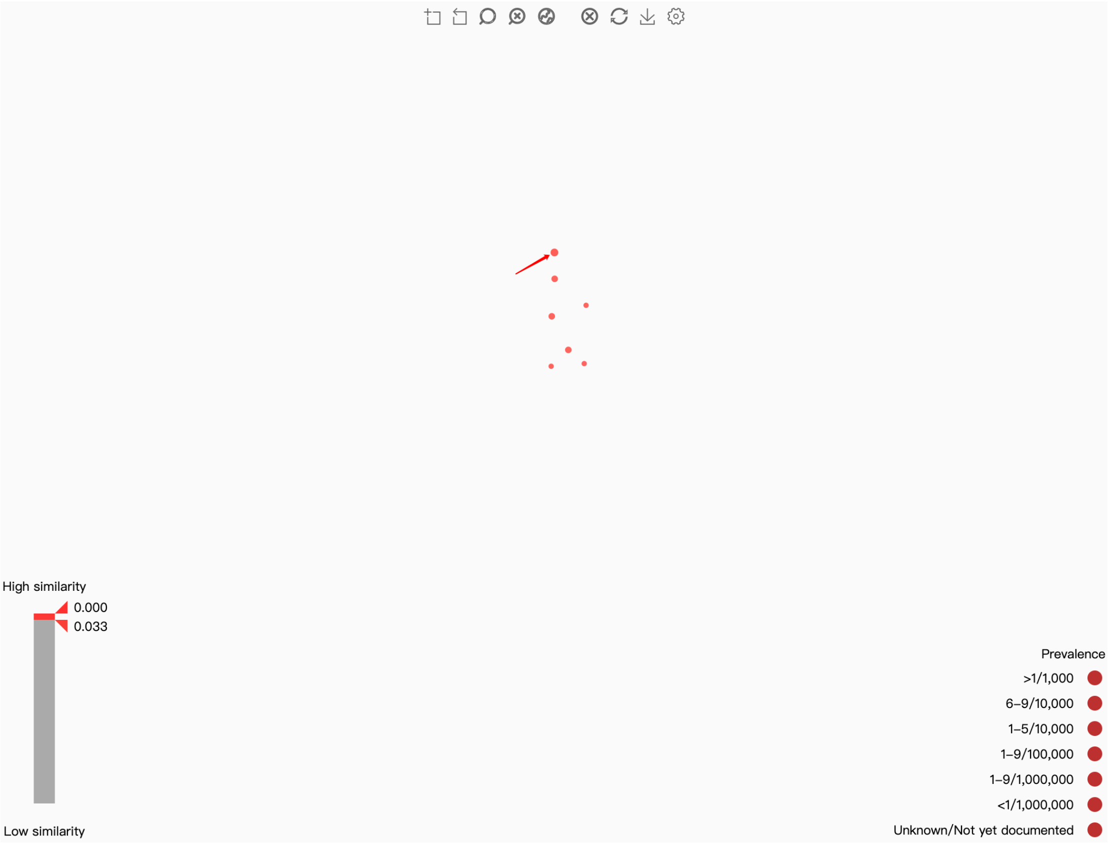

Figure C6-2 The target disease on RDmap of Case6

Case 7:

Publication: Zamel, R., Khan, R., Pollex, R.L. et al. Abetalipoproteinemia: two case reports and literature review. Orphanet J Rare Dis 3, 19 (2008).

Case url: https://ojrd.biomedcentral.com/articles/10.1186/1750-1172-3-19

Diagnosis results: Abetalipoproteinemia

Case presentation:

In 1976, a 16-year old girl presented with progressively worsening coordination. During infancy, her persistent diarrhea resolved with the institution of a low-fat diet. At age 11, routine blood tests revealed acanthocytosis with undetectable plasma cholesterol and triglyceride, which together with normal parental lipid profiles suggested a diagnosis of ABL. Oral vitamin A 25 000 IU daily, 25-hydroxy vitamin D 10 000 IU daily, and vitamin K 5 mg daily were prescribed. At age 16, she began to experience generalized weakness and impaired balance. Neurological examination revealed mild dysarthria, reduced muscle bulk, bilateral proximal muscle weakness, absent deep-tendon reflexes, upgoing plantar reflexes, reduced sensitivity to light touch with loss of proprioception and vibration sense in a glove-and-stocking distribution, mild intention tremor, dysdiadochokinesia in upper and lower limbs, a wide-based ataxic gait and positive Romberg sign.

Test result:

Entered HPO:

HP:0002630; HPO_term: Fat malabsorption; CHPO_term: 脂肪吸收障碍

HP:0001251; HPO_term: Ataxia; CHPO_term: 共济失调

HP:0001324; HPO_term: Muscle weakness; CHPO_term: 肌无力

HP:0001315; HPO_term: Reduced tendon reflexes; CHPO_term: 腱反射减低

Mapping result:

disorder_id	orpha_number	disease_name	distance	
63	550	MELAS（MELAS）	0.0104	
2219	2388	Choreoacanthocytosis（神经棘红细胞增多症）	0.0208	
789	3452	Whipple disease（惠普尔病）	0.0312	No.4
252	14	Abetalipoproteinemia（脂蛋白血症）	0.0416	
12804	96180	Maternal uniparental disomy of chromosome 4（4号染色体母系单亲二体）	0.0416	
112	512	Metachromatic leukodystrophy（变色性白细胞营养不良）	0.0520	
502	2116	Hartnup disease（哈特努普病）	0.0625	

Figure C7-1 Mapping detail of Case7

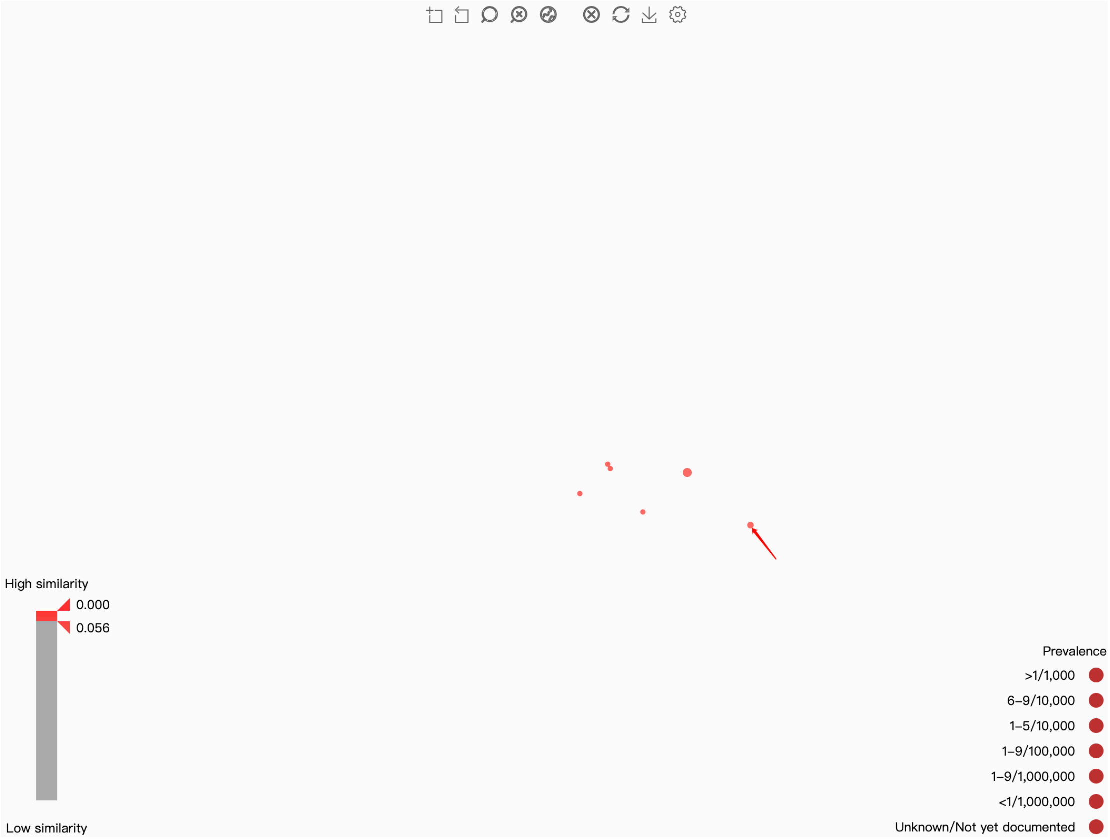

Figure C7-2 The target disease on RDmap of Case7

Case 8:

Publication: Vroegindeweij, L.H.P., Boon, A.J.W., Wilson, J.H.P. et al. Effects of iron chelation therapy on the clinical course of aceruloplasminemia: an analysis of aggregated case reports. Orphanet J Rare Dis 15, 105 (2020).

Case url: https://ojrd.biomedcentral.com/articles/10.1186/s13023-020-01385-w

Diagnosis results: Aceruloplasminemia

Case presentation:

At the time of enrolment, both brothers (case 1, 2) had a two-year history of neurological manifestations and had been treated with phlebotomy monotherapy for several months, which resulted in mildly reduced hemoglobin levels. Both presented initially with normal hemoglobin levels. Their first cousin (case 3) reported stable neurological disease after 13 years of deferoxamine treatment (1000 mg/day, s.c., twice weekly). He was known with microcytic anemia since childhood, which had been treated intermittently with oral iron supplements. At the time of counselling, microcytic anemia was considered to be the first manifestation of aceruloplasminemia in case 3, following routine exclusion of other causes.

Table 1 summarizes the clinical outcomes of treatment with deferiprone and phlebotomy (case 1, 2) and deferiprone in combination with deferoxamine (case 3). All patients deteriorated neurologically, as illustrated by the gradual increase in UPDRS/III and SARA scores over time (Fig. 1). Patient ratings of anxious and depressive feelings (HADS) had only been consistently collected in case 1 and 2, and were highly variable during treatment. Diabetes became manifest despite treatment in case 1 and 3, while in case 2, who was already known with insulin-dependent diabetes at baseline, glucose and HbA1c levels improved and insulin doses could be reduced.

Table 1 Clinical characteristics of our G631R homozygous patients at baseline and end of follow-up

**Case/gender/age[a]**	**Follow-up (months)**	**Neurological function**		**Retinopathy**		**Diabetes**		**Anemia**	
		**Baseline**	**EFU**	**Baseline**	**EFU**	**Baseline**	**EFU**	**Baseline**	**EFU**
1/M/50	18	Orofacial dyskinesia, chorea, dystonia, dysarthria, gait disturbance	Died	No	NA	No	Yes	Yes[b]	Yes
2/M/56	76	Dysarthria, ataxia, gait disturbance, behavioral changes	Progressive speech and gait disturbance, wheelchair-bound, aspiration, cognitive decline and apathy	No	No	Yes	Yes	Yes[b]	No
3/M/61	70	Stress related tremor, slightly diminished	Development of gait disturbance with falls, mental	No	No	No	Yes	Yes	Yes

Case/gender/age[a]	Follow-up (months)	Neurological function		Retinopathy		Diabetes		Anemia	
		Baseline	EFU	Baseline	EFU	Baseline	EFU	Baseline	EFU
		facial expression	slowing, behavioral changes						

Test result:

Entered HPO:

HP:0001935; HPO_term: Microcytic anemia; CHPO_term: 小细胞性贫血

HP:0001260; HPO_term: Dysarthria; CHPO_term: 构音障碍

HP:0001288; HPO_term: Gait disturbance; CHPO_term: 步态不稳

HP:0000819; HPO_term: Diabetes mellitus; CHPO_term: 糖尿病

HP:0001903; HPO_term: Anemia; CHPO_term: 贫血

HP:0001300; HPO_term: Parkinsonism; CHPO_term: 帕金森症

Mapping result:

No.1

disorder_id	orpha_number	disease_name	distance
10633	48818	Aceruloplasminemia（无铜蓝蛋白血症）	0.0416
14707	100996	Autosomal recessive spastic paraplegia type 15（常染色体隐性痉挛性截瘫15型）	0.0625
812	3463	Wolfram syndrome（Wolfram综合征）	0.0625
2219	2388	Choreoacanthocytosis（神经棘红细胞增多症）	0.0694
644	355	Gaucher disease（戈谢病）	0.0763
2480	2719	Oculocerebral hypopigmentation syndrome, Cross type（眼脑色素减退综合征，交叉型）	0.0763

Figure C8-1 Mapping detail of Case8

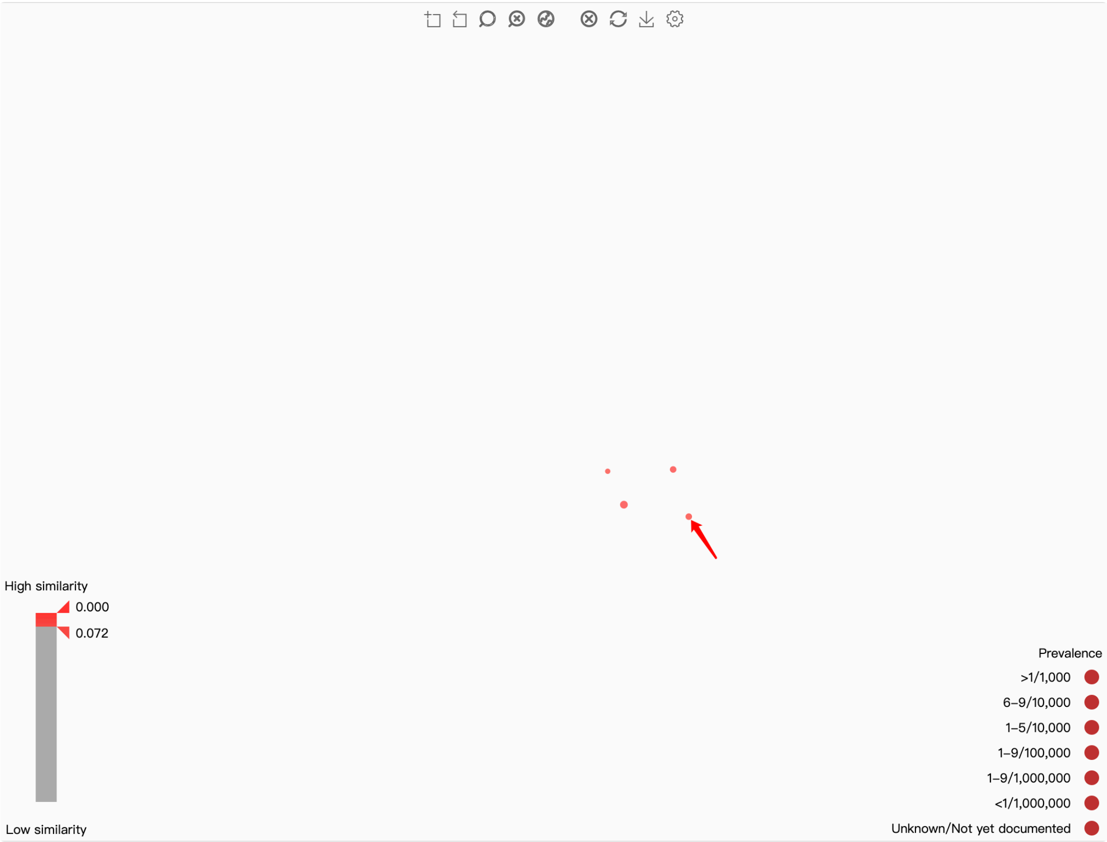

Figure C8-2 The target disease on RDmap of Case8

Case 9:

Publication: Zhou, L., Ouyang, R., Luo, H. et al. Efficacy of sirolimus for the prevention of recurrent pneumothorax in patients with lymphangioleiomyomatosis: a case series. Orphanet J Rare Dis 13, 168 (2018).

Case url: https://ojrd.biomedcentral.com/articles/10.1186/s13023-018-0915-2

Diagnosis results: Lymphangioleiomyomatosis

Case presentation:

A 33-year-old female nonsmoker with a 4-month history of intermittent chest pain and dyspnea at rest, which recurred every 2 weeks, was admitted to our hospital at 31 weeks of gestation. Four months ago, she had been admitted after experiencing these symptoms for the first time. A chest radiograph at that time revealed left hydropneumothorax with 90% lung compression. The patient received closed chest tube drainage (CTD). However, left pneumothorax recurred during rest or minimal activity in the 20th, 25th, 28th, and 30th weeks of gestation.

Test result:

Entered HPO:

HP:0100749; HPO_term: Chest pain; CHPO_term: 胸痛

HP:0002094; HPO_term: Dyspnea; CHPO_term: 呼吸困难

HP:0002107; HPO_term: Pneumothorax; CHPO_term: 气胸

Mapping result:

disorder_id	orpha_number	disease_name	distance	No.1
3386	538	Lymphangioleiomyomatosis（淋巴管平滑肌瘤病）	0.0	
14494	99921	Chronic graft versus host disease（慢性移植物抗宿主病）	0.0	
735	797	Sarcoidosis（结节病）	0.0	
12138	91387	Familial thoracic aortic aneurysm and aortic dissection（家族性胸主动脉瘤和主动脉夹层）	0.0138	
10645	50251	Pleural mesothelioma（胸膜间皮瘤）	0.0138	
10845	64741	Pulmonary blastoma（肺母细胞瘤）	0.0277	

Figure C9-1 Mapping detail of Case9

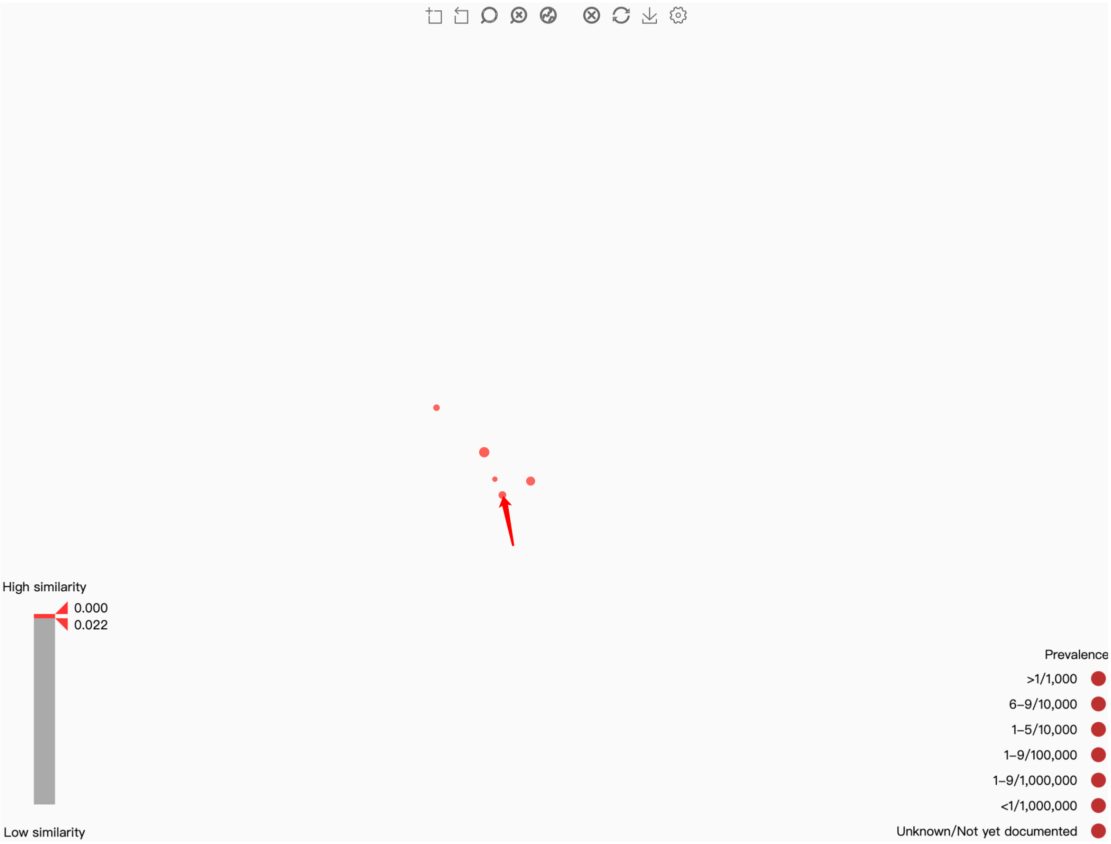

Figure C9-2 The target disease on RDmap of Case9

Case 10:

Publication: Dias, R.P., Buchanan, C.R., Thomas, N. et al. Os odontoideum in wolcott-rallison syndrome: a case series of 4 patients. Orphanet J Rare Dis 11, 14 (2016).

Case url: https://ojrd.biomedcentral.com/articles/10.1186/s13023-016-0397-z

Diagnosis results: Wolcott-Rallison syndrome

Case presentation:

This female patient, now aged 21 years, presented with antibody negative (Islet Cell / Glutamic Acid decarboxylase) diabetes mellitus aged 6 months. Her parents are White British and unrelated. At 3 years of age, she developed acute liver failure, encephalopathy and renal dysfunction during a minor febrile illness. She recovered completely except for residual cerebellar signs (dysarthria, ataxia). Progressive growth failure developed from 3–4 years of age prompting assessment of pituitary function which did not reveal any abnormality. Hand radiography revealed acro-osteolysis of distal phalanges 1,3 and 5 and the dysplastic carpal bones. Subsequent skeletal survey confirmed multiple epiphyseal dysplasia with hip subluxation and extensive calcification of soft tissues around the knee joints. WRS was confirmed on genetic testing which revealed compound heterozygosity for a frameshift mutation c.577delA and a missense mutation R632W (c.1966C > T) in exons 3 and 12 of the E1F2AK3 gene.

Test result:

Entered HPO:

HP:0006554; HPO_term: Acute hepatic failure; CHPO_term: 急性肝功能衰竭

HP:0001298; HPO_term: Encephalopathy; CHPO_term: 脑病

HP:0000083; HPO_term: Renal insufficiency; CHPO_term: 肾功能不全

HP:0002654; HPO_term: Multiple epiphyseal dysplasia; CHPO_term: 多发性骨骺发育不良

Mapping result:

disorder_id	orpha_number	disease_name	distance
1754	1667	Wolcott-Rallison syndrome（沃尔科特-雷利森综合征）	0.0208
876	397	Giant cell arteritis（巨细胞动脉炎）	0.0729
21628	319218	Ebola hemorrhagic fever（埃博拉出血热）	0.0729
14402	99829	Yellow fever（黄热病）	0.0729
8539	108	Babesiosis（幼虫病）	0.0833
355	352	Galactosemia（半乳糖血症）	0.0833
1007	528	Berardinelli-Seip congenital lipodystrophy（Berardinelli-Seip先天性脂肪营养不良）	0.0833

No.1

Figure C10-1 Mapping detail of Case10

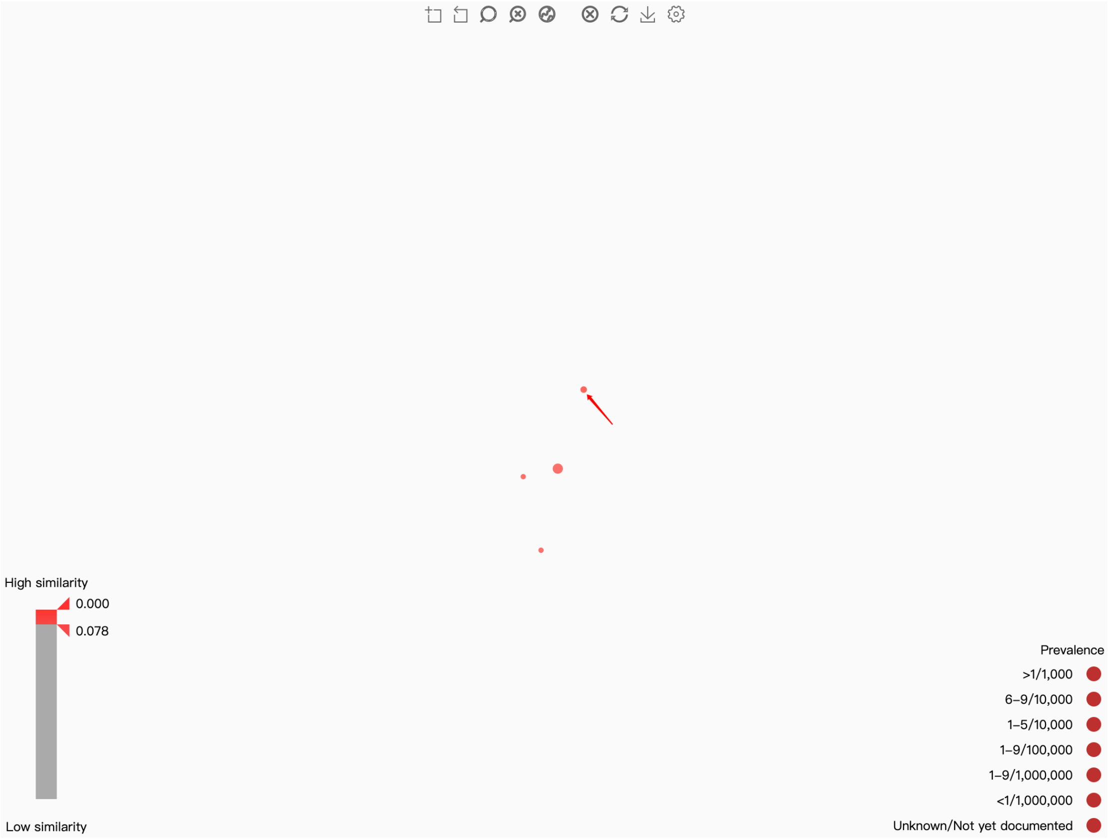

Figure C10-2 The target disease on RDmap of Case10

Case 11:

Publication: Valayannopoulos, V., Nicely, H., Harmatz, P. et al. Mucopolysaccharidosis VI. Orphanet J Rare Dis 5, 5 (2010).

Case url: https://ojrd.biomedcentral.com/articles/10.1186/1750-1172-5-5

Diagnosis results: Mucopolysaccharidosis type 6

Case presentation:

Rapidly progressing 16-year old MPS VI patient: Photograph of face showing coarse facies: frontal bossing, enlarged tongue, thick lips, abnormal dentition and gingival hyperplasia. Photograph of patient showing curvature of spine (lumbar kyphosis, scoliosis, lordosis).

Test result:

Entered HPO:

HP:0000280; HPO_term: Coarse facial features; CHPO_term: 面容粗糙

HP:0000470; HPO_term: Short neck; CHPO_term: 短颈

HP:0000158; HPO_term: Macroglossia; CHPO_term: 巨舌症

HP:0002808; HPO_term: Kyphosis; CHPO_term: 脊柱后凸畸形（驼背）

HP:0012471; HPO_term: Thick vermilion border; CHPO_term: 厚嘴唇

Mapping result:

disorder_id	orpha_number	disease_name	distance
131	580	Mucopolysaccharidosis type 2（黏多醣贮积症2型）	0.0083
24	583	Mucopolysaccharidosis type 6（黏多醣贮积症6型）	0.0083
12381	93473	Hurler syndrome（赫勒综合征）	0.0166
145	904	Williams syndrome（威廉姆斯综合症）	0.0166
19869	261290	Trisomy 17p（三体17p）	0.0166
19876	261337	Distal 22q11.2 microduplication syndrome（远端22q11.2微复制综合征）	0.0333
3	61	Alpha-mannosidosis（α-甘露糖苷病）	0.0333

No.1

Figure C11-1 Mapping detail of Case11

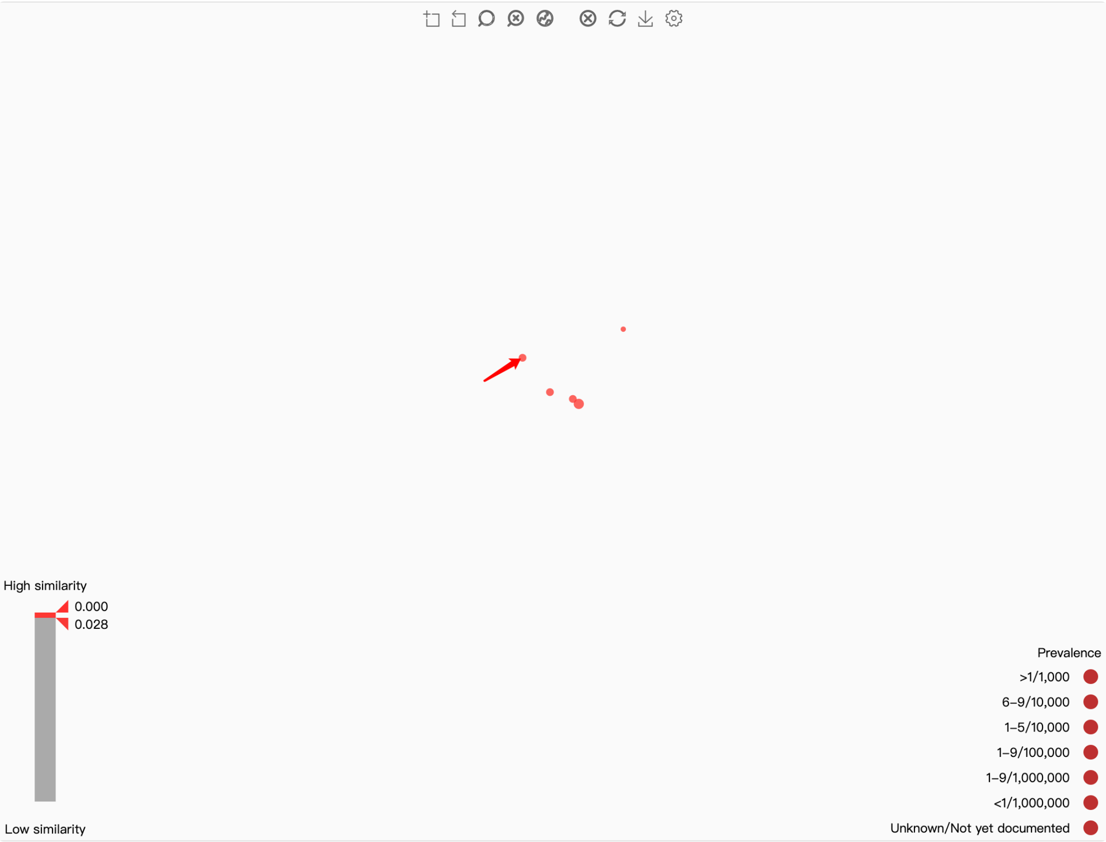

Figure C11-2 The target disease on RDmap of Case11

Case 12:

Publication: Biesecker, L.G. The Greig cephalopolysyndactyly syndrome. Orphanet J Rare Dis 3, 10 (2008).

Case url: https://ojrd.biomedcentral.com/articles/10.1186/1750-1172-3-10

Diagnosis results: Greig cephalopolysyndactyly syndrome

Case presentation:

A patient with Greig cephalopolysyndactyly. A. Facial view of the patient. Note the hypertelorism and macrocephaly. B. The hand of this patient shows a broad thumb, complete cutaneous syndactyly of digits 2–5 with fusion of the nails and a postaxial supernumerary digit. C. The foot of this patient shows a partially duplicated hallux with cutaneous syndactyly of several digits.

Test result:

Entered HPO:

HP:0000256; HPO_term: Macrocephaly; CHPO_term: 巨头畸形

HP:0011304; HPO_term: Broad thumb; CHPO_term: 拇指变宽

HP:0001159; HPO_term: Syndactyly; CHPO_term: 并指（趾）畸形

HP:0001162; HPO_term: Postaxial hand polydactyly; CHPO_term: 轴后多指畸形

HP:0005873; HPO_term: Polysyndactyly of hallux; CHPO_term: 拇趾多指

Mapping result:

disorder_id	orpha_number	disease_name	distance	No.1
2011	380	Greig cephalopolysyndactyly syndrome（格雷格头足综合征）	0.016	
10879	65759	Carpenter syndrome（Carpenter综合症）	0.032	
2130	672	Pallister-Hall syndrome（Pallister-Hall综合征）	0.04	
912	373	Simpson-Golabi-Behmel syndrome（辛普森-戈拉比-贝梅尔综合征）	0.04	
19598	250989	1q21.1 microdeletion syndrome（1q21.1微缺失综合征）	0.064	
1674	1507	Autosomal recessive Robinow syndrome（常染色体隐性Robinow综合征）	0.064	

Figure C12-1 Mapping detail of Case12

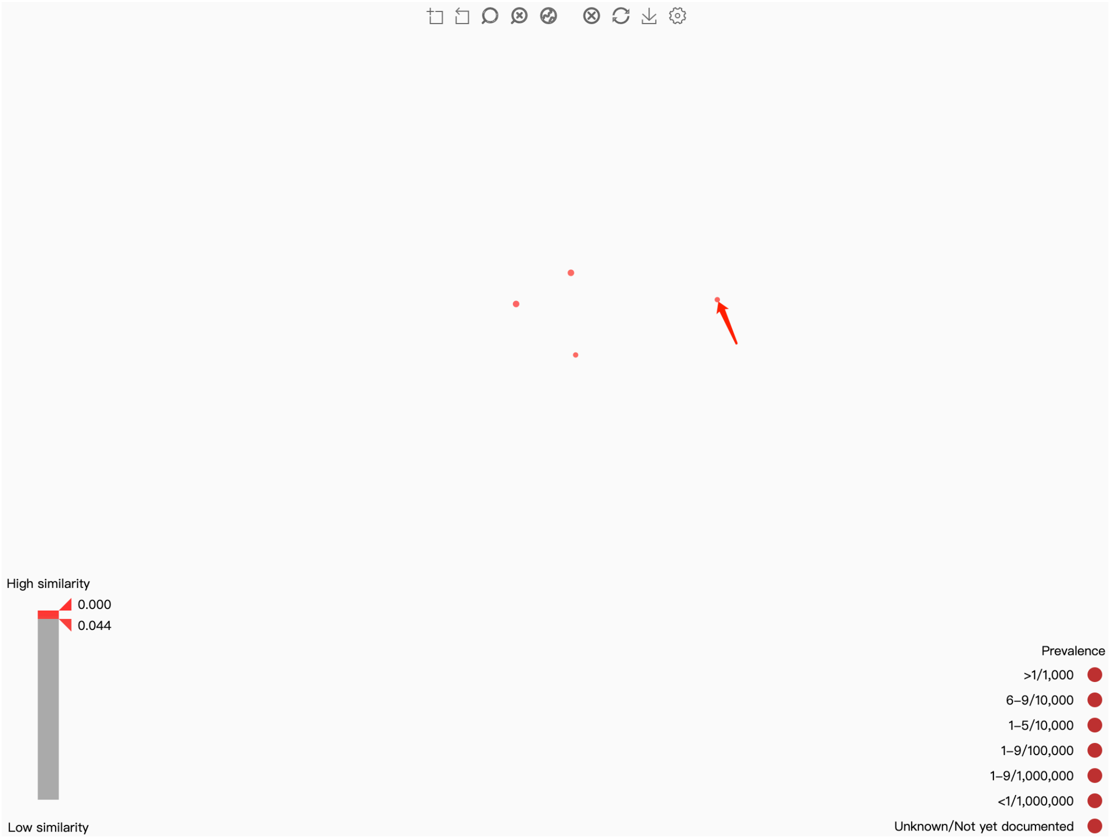

Figure C12-2 The target disease on RDmap of Case12

Case 13:

Publication: Germain, D.P. Fabry disease. Orphanet J Rare Dis 5, 30 (2010).

Case url: https://ojrd.biomedcentral.com/articles/10.1186/1750-1172-5-30

Diagnosis results: Fabry disease

Case presentation:

Angiokeratoma: the angiokeratoma are small, raised, dark-red spots that increase in number and size with age and can occur singly or in clusters. They are typically found on the lower back, buttocks, groin, flanks and upper thighs but their distribution may be restricted to a limited area, such as the umbilicus.

Test result:

Entered HPO:

HP:0001014; HPO_term: Angiokeratoma; CHPO_term: 血管角皮瘤

Mapping result:

disorder_id	orpha_number	disease_name	distance
94	324	Fabry disease（法布里病）	0.0
10743	53721	Cobb syndrome（科布综合征）	0.0
18935	221061	Familial cerebral cavernous malformation（家族性脑海绵状畸形）	0.0416
5	93	Aspartylglucosaminuria（天冬氨酰葡糖胺尿症）	0.0416
20500	280779	Cutaneous collagenous vasculopathy（皮肤胶原性血管病）	0.0416
10742	53719	Wyburn-Mason syndrome（Wyburn-Mason综合征）	0.0416
26	812	Sialidosis type 1（涎酸累积症1型）	0.0416

No.1

Figure C13-1 Mapping detail of Case13

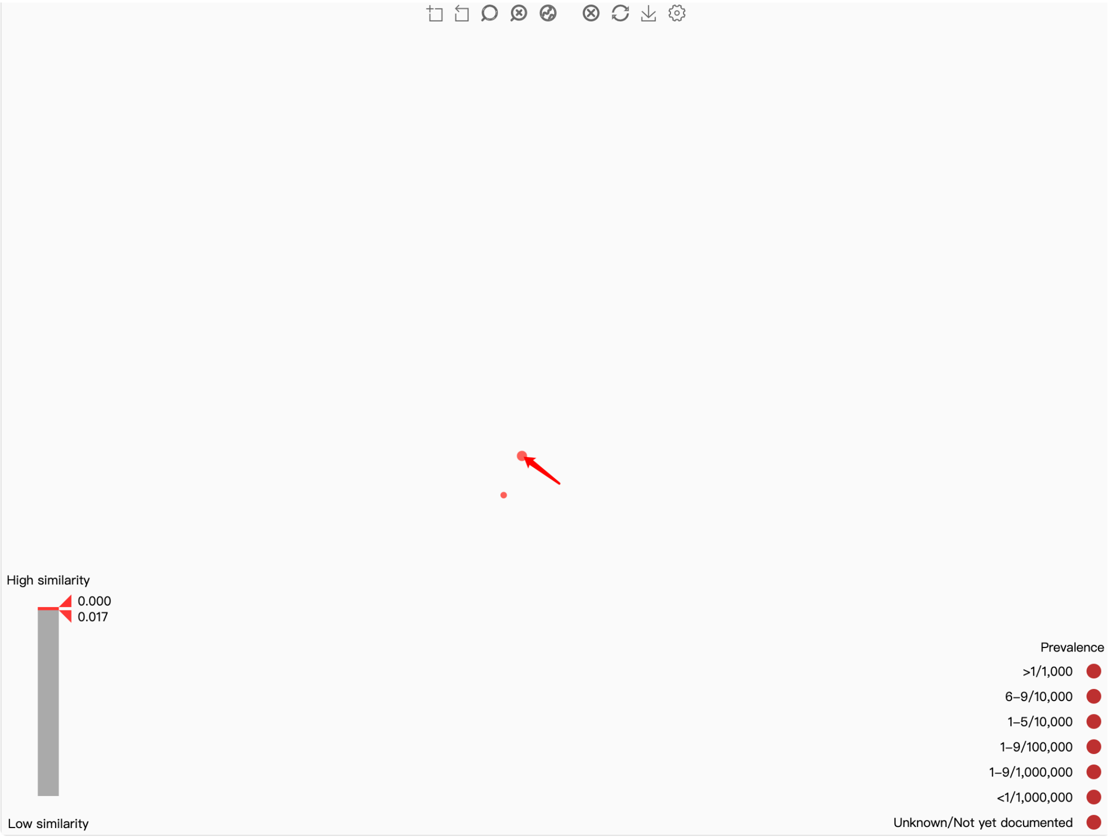

Figure C13-2 The target disease on RDmap of Case13

Case 14:

Publication: Drera, B., Ritelli, M., Zoppi, N. et al. Loeys-Dietz syndrome type I and type II: clinical findings and novel mutations in two Italian patients. Orphanet J Rare Dis 4, 24 (2009).

Case url: https://ojrd.biomedcentral.com/articles/10.1186/1750-1172-4-24

Diagnosis results: Loeys-Dietz syndrome

Case presentation:

Patient 1 was a seven years old male, born at term after uncomplicated pregnancy from non-consanguineous parents. At birth, weight and length were normal; he showed bilateral clubfoot and hand contractures characterized by camptodactyly and ulnar deviation. At the age of six years he showed: height 130.5 cm, arm span 129 cm, upper:lower segment ratio 0.89, muscular hypotrophy; dolicocephaly, blue sclerae, hypoplastic alae nasi, microretrognathia, high-arched palate, bifid uvula; mild thoracic scoliosis and pectus excavatum, pes planus, joint hypermobility (Beighton score 8/9); velvety and translucent skin (Fig. 1A). Echocardiogram showed bicuspid aortic valve, patent foramen ovale, mild interventricular septal hypertrophy without outflow obstruction, mild mitral valve prolapse, aortic root diameter 23 mm and ascending aorta diameter 21 mm. Magnetic Resonance Angiography (MRA) displayed tortuosity and elongation of the basilar and internal carotid arteries with kinking and coiling of these last. Furthermore, the patient suffered from allergic asthma and atopic dermatitis.

Test result:

Entered HPO:

HP:0100490; HPO_term: Camptodactyly of finger; CHPO_term: 手指弯曲

HP:0001193; HPO_term: Ulnar deviation of the hand or of fingers of the hand; CHPO_term: 手或手指尺侧偏斜

HP:0001776; HPO_term: Bilateral talipes equinovarus; CHPO_term: 双侧马蹄内翻足

HP:0000592; HPO_term: Blue sclerae; CHPO_term: 蓝巩膜

HP:0000308; HPO_term: Microretrognathia; CHPO_term: 小下颌后移

HP:0000218; HPO_term: High palate; CHPO_term: 高腭

HP:0000193; HPO_term: Bifid uvula; CHPO_term: 双歧悬雍垂

Mapping result:

disorder_id	orpha_number	disease_name	distance
10799	60030	Loeys-Dietz syndrome（Loeys-Dietz综合征）	0.0628
14349	99776	Mosaic trisomy 9（Mosaic三体性9）	0.0742
1388	1106	Microphthalmia with limb anomalies（伴有肢体异常的小眼症）	0.08
1674	1507	Autosomal recessive Robinow syndrome（常染色体隐性Robinow综合征）	0.08
3191	1101	Anophthalmia-megalocornea-cardiopathy-skeletal anomalies syndrome（无眼症-大眼角膜炎-心脏病-骨骼异常综合征）	0.0857

No.1

Figure C14-1 Mapping detail of Case14

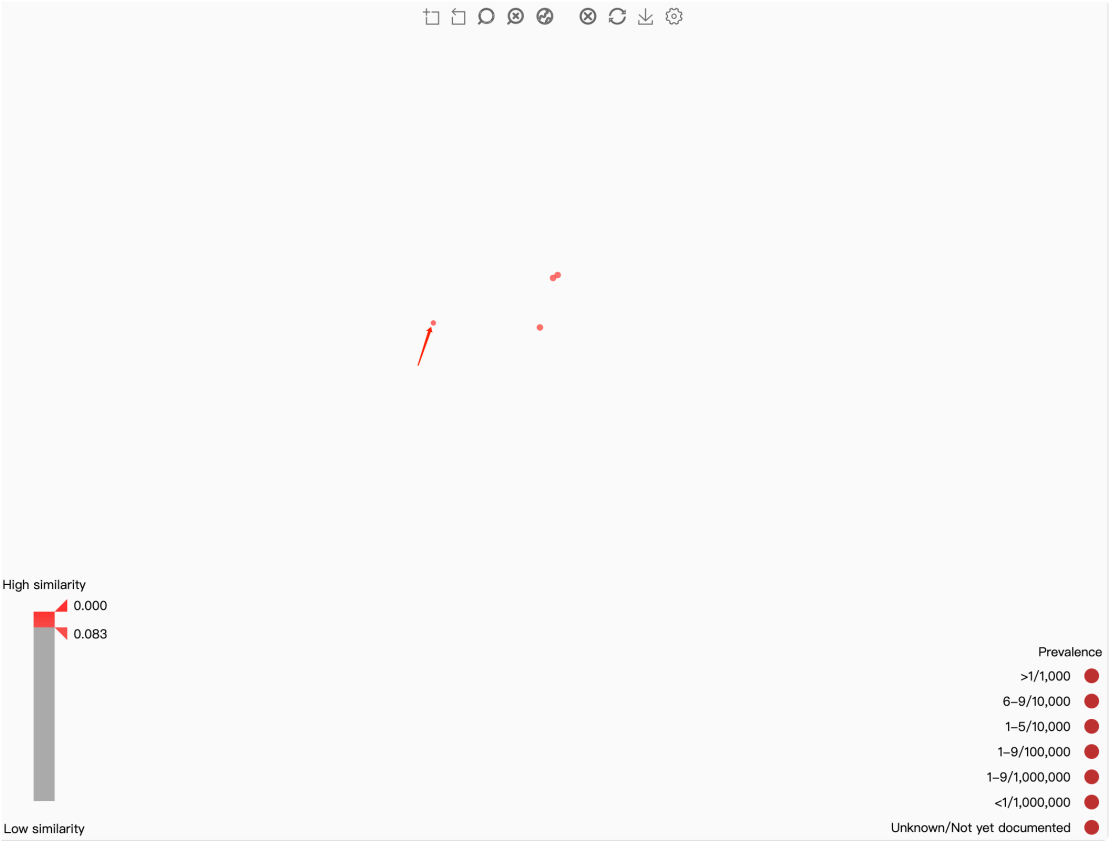

Figure C14-2 The target disease on RDmap of Case14

Case 15:

Publication: Reibel, A., Manière, M., Clauss, F. et al. Orodental phenotype and genotype findings in all subtypes of hypophosphatasia. Orphanet J Rare Dis 4, 6 (2009).

Case url: https://ojrd.biomedcentral.com/articles/10.1186/1750-1172-4-6

Diagnosis results: Hypophosphatasia

Case presentation:

The boy was referred to the Paediatric Department of the Hôpitaux Universitaires de Strasbourg at 9 years of age. He has the infantile form of HP which was diagnosed at birth. He had experienced multiple fractures and craniosynostosis. Currently, he is confined to a wheelchair. Screening for the HP mutations was performed to confirm the diagnosis. At 9 years of age, he has already lost all mandibular primary teeth except the right lower first molar (84). The maxillary primary central incisors and second primary molars (51, 61, 55 and 65) had also been prematurely exfoliated. Currently, the patient is in the mixed dentition stage. The primary teeth still present are the maxillary lateral incisors, the maxillary canines, the maxillary first molars and the right lower first molar (52, 53, 54, 62, 63, 64 and 84), the permanent teeth present are the maxillary left central incisor, all four lower incisors and all first molars (21, 31, 32, 41, 42, 16, 26, 36 and 46).

Test result:

Entered HPO:

HP:0002757; HPO_term: Recurrent fractures; CHPO_term: 复发性骨折

HP:0001363; HPO_term: Craniosynostosis; CHPO_term: 颅缝早闭

HP:0006480; HPO_term: Premature loss of teeth; CHPO_term: 牙齿过早脱落

Mapping result:

disorder_id	orpha_number	disease_name	distance
839	2314	Autosomal dominant hyper-IgE syndrome（常染色体显性遗传性高IgE综合征）	0.0138
2530	667	Autosomal recessive malignant osteopetrosis（常染色体隐性恶性骨质疏松症）	0.0138
162	436	Hypophosphatasia（低磷酸脂酶症）	0.0138
3700	2781	Osteopetrosis and related disorders（骨质疏松症及相关疾病）	0.0138
11911	89936	X-linked hypophosphatemia（X连锁低磷血症）	0.0138
654	666	Osteogenesis imperfecta（成骨不全症）	0.0416

No.1

Figure C15-1 Mapping detail of Case15

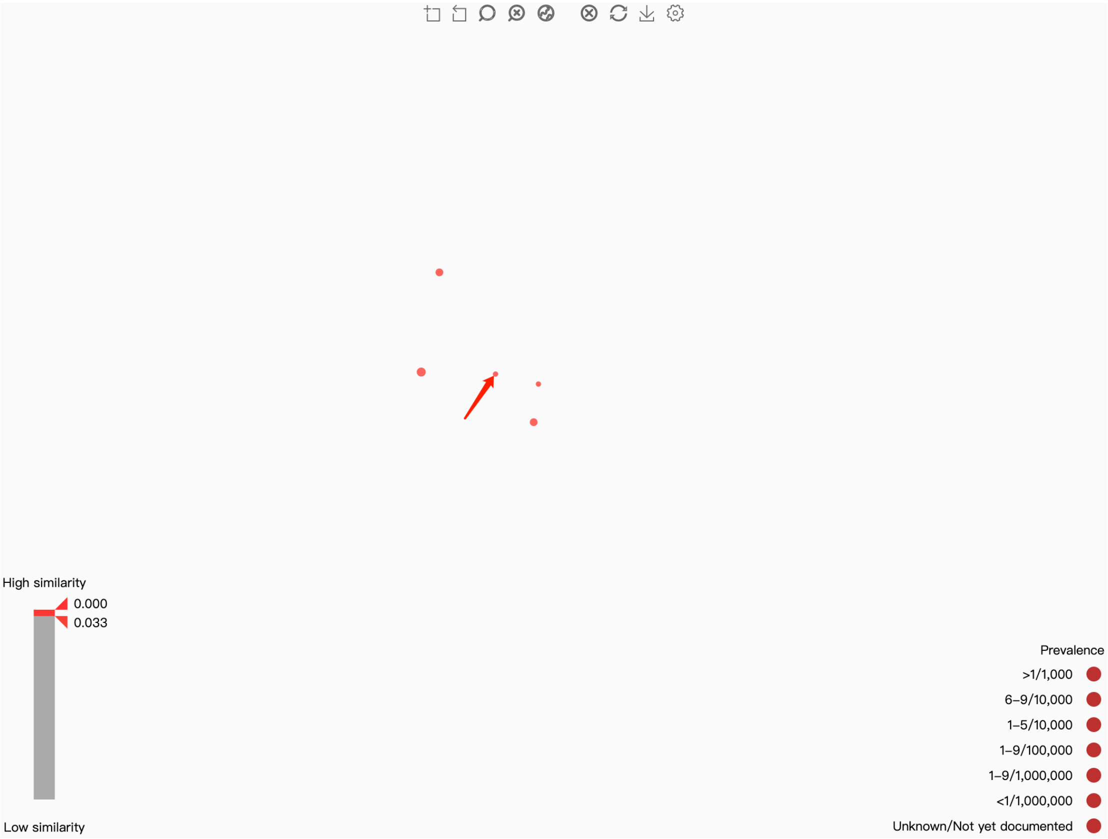

Figure C15-2 The target disease on RDmap of Case15

Case 16:

Publication: Sarfati, J., Bouvattier, C., Bry-Gauillard, H. et al. Kallmann syndrome with FGFR1 and KAL1 mutations detected during fetal life. Orphanet J Rare Dis 10, 71 (2015).

Case url: https://ojrd.biomedcentral.com/articles/10.1186/s13023-015-0287-9

Diagnosis results: Kallmann syndrome

Case presentation:

The proband of this family was a 32-year-old woman who consulted for infertility. She had breast development at the age of 12.5 years and menarche at 14.5 years. On consulting for oligomenorrhea (4 menstrual bleeds per year) at the age of 18, she was prescribed a combined oral contraceptive, without an etiological work-up, and subsequently had regular menses. Wishing to become pregnant, she stopped using contraception at 29 years of age. Oligomenorrhea recurred and she failed to conceive despite regular intercourse and her partner's normal sperm count. She then consulted a gynecologist who prescribed her clomiphene citrate. After four rounds of treatment, which led to neither ovulation nor pregnancy, she was referred to our department for an Endocrine consultation. During the interview she noted no sense of smell (anosmia) that was also present in her maternal grandfather. In addition, she reported poor hearing as well as absent premolars and wisdom teeth.

Test result:

Entered HPO:

HP:0000876; HPO_term: Oligomenorrhea; CHPO_term: 月经稀发

HP:0003187; HPO_term: Breast hypoplasia; CHPO_term: 乳房发育不良

HP:0000458; HPO_term: Anosmia; CHPO_term: 嗅觉丧失

HP:0000365; HPO_term: Hearing impairment; CHPO_term: 听力障碍

HP:0009804; HPO_term: Reduced number of teeth; CHPO_term: 牙齿数量减少

Mapping result:

disorder_id	orpha_number	disease_name	distance	No.1
3249	478	Kallmann syndrome（卡门氏症候群）	0.0249	
542	570	Moebius syndrome（莫比乌斯综合征）	0.0666	
110	138	CHARGE syndrome（CHARGE综合征）	0.0916	
8668	432	Normosmic congenital hypogonadotropic hypogonadism（正常先天性性腺功能减退性腺功能减退症）	0.0916	
1247	920	Ablepharon macrostomia syndrome（大肌麻痹综合征）	0.0999	
14199	99226	Monosomy X（单体X）	0.0999	
44	881	Turner syndrome（特纳综合征）	0.0999	

Figure C16-1 Mapping detail of Case16

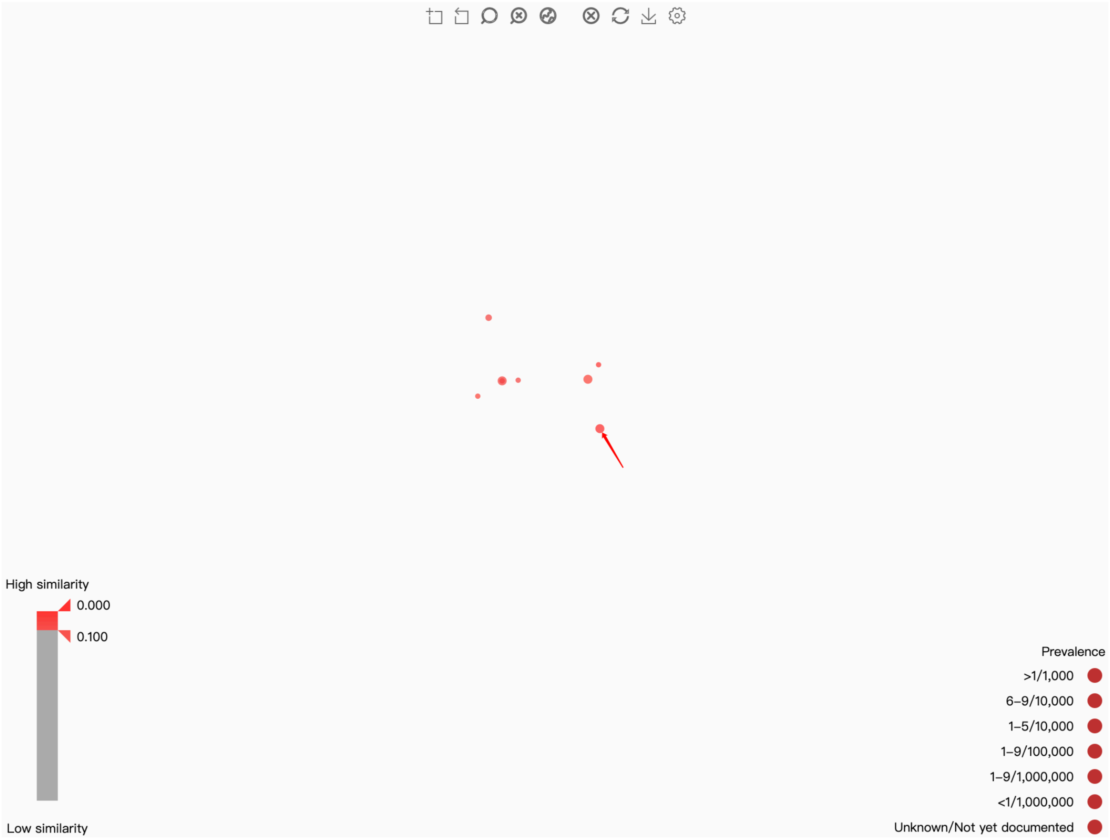

Figure C16-2 The target disease on RDmap of Case16

Case 17:

Publication: Weisfeld-Adams, J.D., Mehta, L., Rucker, J.C. et al. Atypical Chédiak-Higashi syndrome with attenuated phenotype: three adult siblings homozygous for a novel LYST deletion and with neurodegenerative disease. Orphanet J Rare Dis 8, 46 (2013).

Case url: https://ojrd.biomedcentral.com/articles/10.1186/1750-1172-8-46

Diagnosis results: Chédiak-Higashi syndrome

Case presentation:

Three affected siblings were part of a large consanguineous family of Pakistani extraction. The proband (III:9), was 40 years old at the time of initial evaluation in Medical Genetics. She had a 9 year history of progressive leg weakness with cognitive impairment, cerebellar ataxia with hypermetric saccades, and parkinsonian features including bradykinesia, masked facies, and hypophonia. In childhood, she had a history of learning difficulties and attention-deficit behaviors without hyperactivity. She had a long history of arthralgias and limb contractures. She had generalized hyperpigmentation, noted in infancy and followed by the appearance of widespread hypopigmented macules persisting into adulthood. She had a history of multiple dental infections in childhood and severe periodontitis at the age of 36 years, but no other history suggestive of immunodeficiency.

Test result:

Entered HPO:

HP:0007340; HPO_term: Lower limb muscle weakness; CHPO_term: 下肢肌肉无力

HP:0000726; HPO_term: Dementia; CHPO_term: 认知障碍

HP:0001251; HPO_term: Ataxia; CHPO_term: 共济失调

HP:0007338; HPO_term: Hypermetric saccades; CHPO_term: Hypermetric扫视

HP:0002067; HPO_term: Bradykinesia; CHPO_term: 运动迟缓

HP:0000704; HPO_term: Periodontitis; CHPO_term: 牙周炎

Mapping result:

disorder_id	orpha_number	disease_name	distance
63	550	MELAS（MELAS）	0.0833
14707	100996	Autosomal recessive spastic paraplegia type 15（常染色体隐性痉挛性截瘫15型）	0.0902
3253	910	Xeroderma pigmentosum（色素性干皮病）	0.0902
249	167	Chédiak-Higashi syndrome（Chédiak-Higashi综合征）	0.0972
19795	254886	Autosomal recessive progressive external ophthalmoplegia（常染色体隐性进行性眼外肌麻痹）	0.0972
21249	306674	Kufor-Rakeb syndrome（Kufor-Rakeb综合征）	0.0972

No.4

Figure C17-1 Mapping detail of Case17

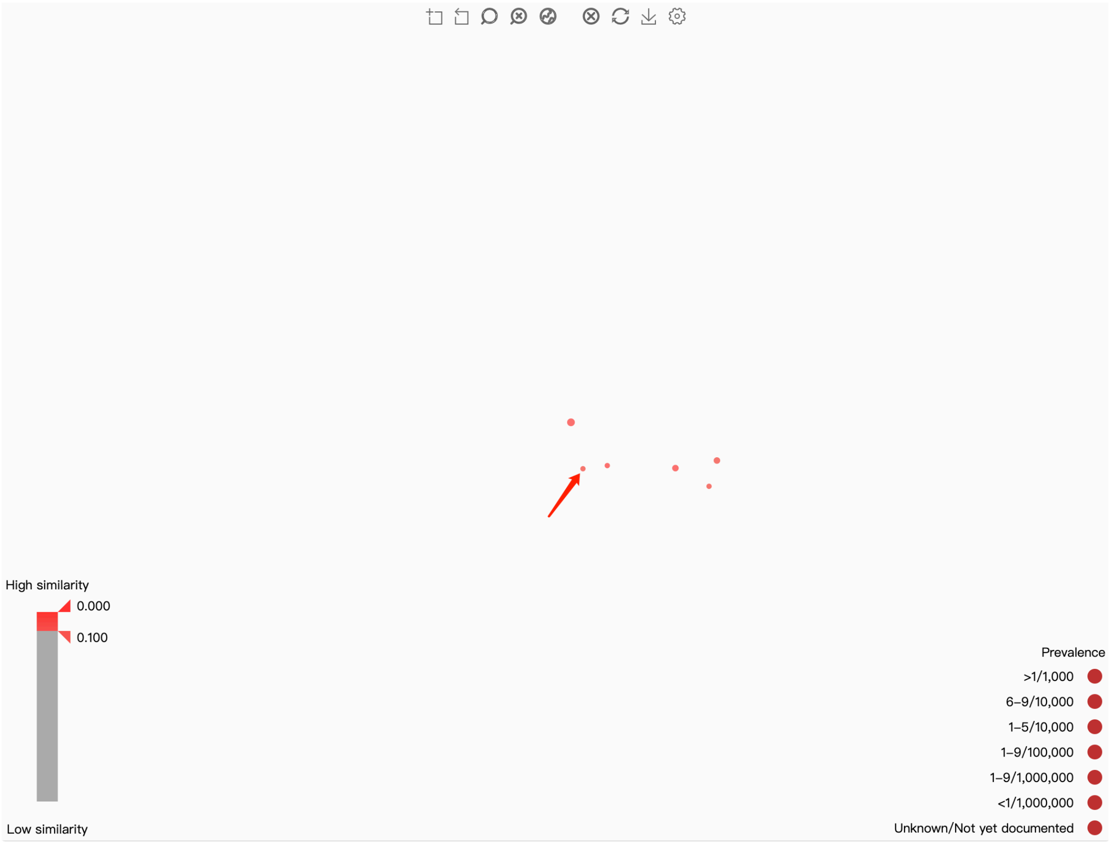

Figure C17-2 The target disease on RDmap of Case17

Case 18:

Publication: Garavelli, L., Mainardi, P.C. Mowat-Wilson syndrome. Orphanet J Rare Dis 2, 42 (2007).

Case url: https://ojrd.biomedcentral.com/articles/10.1186/1750-1172-2-42

Diagnosis results: Mowat-Wilson syndrome

Case presentation:

In infancy, there are excess nuchal skin, a rounded skull shape, a sparse fine hair and a puffy anterior neck; the face is square shaped with high forehead, frontal bossing, hypertelorism, strabismus, epicanthus, deep set but large eyes, a broad nasal bridge, saddle nose with prominent rounded nasal tip, prominent columella, open mouth, with M-shaped upper lip, frequent smiling, and a prominent but narrow and triangular pointed chin. Additional suggestive facial features include telecanthus, a full or everted lower lip and posteriorly rotated ears. The more consistent and easily recognisable features are the eyebrows, which are large, medially flaring and sparse in the middle part and the ear lobes, which are very typical. They are large and uplifted with a central depression and have been described as being like "orecchiette pasta" or like "red blood corpuscles" in shape.

Test result:

Entered HPO:

HP:0000194; HPO_term: Open mouth; CHPO_term: 张口

HP:0000534; HPO_term: Abnormality of the eyebrow; CHPO_term: 眉毛异常

HP:0002007; HPO_term: Frontal bossing; CHPO_term: 前额突出

HP:0000490; HPO_term: Deeply set eye; CHPO_term: 眼睛深陷

HP:0000431; HPO_term: Wide nasal bridge; CHPO_term: 宽鼻梁

HP:0000486; HPO_term: Strabismus; CHPO_term: 斜视

Mapping result:

disorder_id	orpha_number	disease_name	distance	No.1
2051	2152	Mowat-Wilson syndrome（莫瓦威尔逊综合征）	0.0	
387	819	Smith-Magenis syndrome（Smith-Magenis综合征）	0.0069	
1738	1606	1p36 deletion syndrome（1p36缺失综合征）	0.0277	
1683	1516	Craniofacial dyssynostosis（颅面骨不连）	0.0347	
147	280	Wolf-Hirschhorn syndrome（Wolf-Hirschhorn综合征）	0.0347	
19859	261211	16p11.2p12.2 microdeletion syndrome（16p11.2p12.2微缺失综合征）	0.0347	

Figure 18-1 Mapping detail of Case18

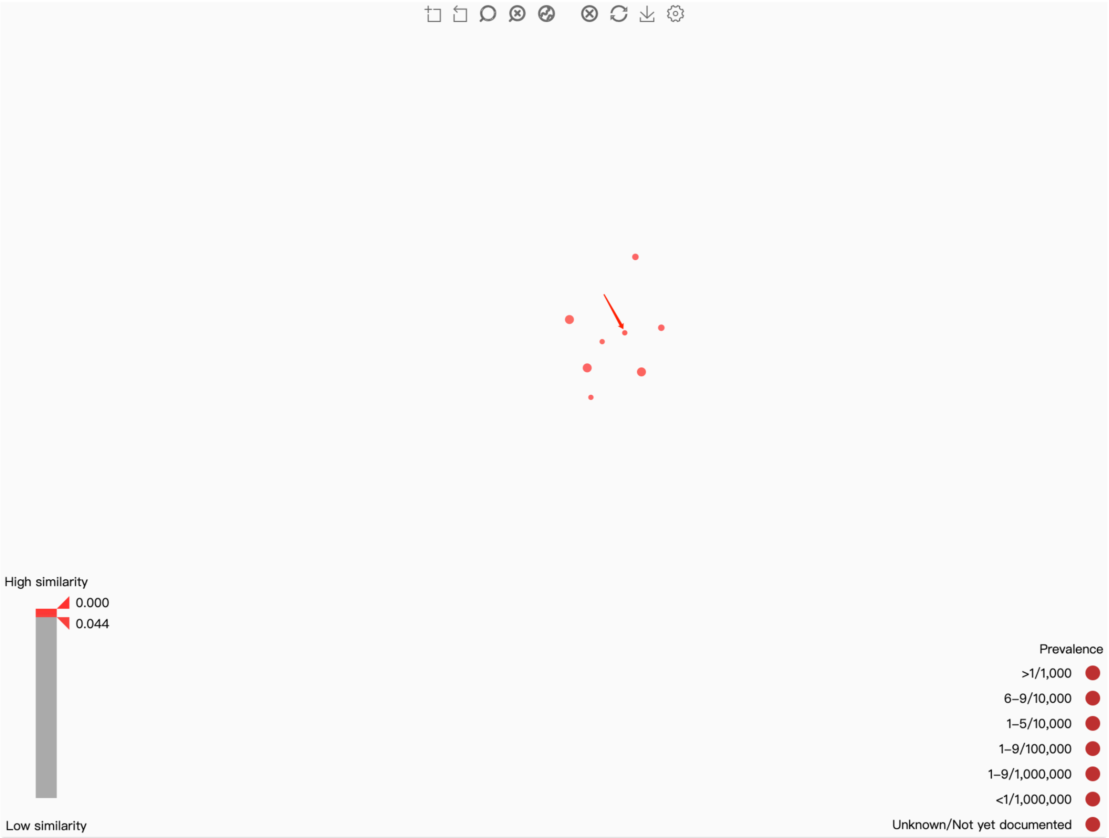

Figure C18-2 The target disease on RDmap of Case18

Case 19:

Publication: Chrzanowska, K.H., Gregorek, H., Dembowska-Bagińska, B. et al. Nijmegen breakage syndrome (NBS). Orphanet J Rare Dis 7, 13 (2012).

Case url: https://ojrd.biomedcentral.com/articles/10.1186/1750-1172-7-13

Diagnosis results: Nijmegen breakage syndrome

Case presentation:

Facial phenotype in NBS: face and profile of a girl aged 3.5 years. Note microcephaly, sloping forehead, small nose, receding chin, and relatively large ears.

Test result:

Entered HPO:

HP:0000252; HPO_term: Microcephaly; CHPO_term: 小头畸形

HP:0000340; HPO_term: Sloping forehead; CHPO_term: 额头倾斜

HP:0000278; HPO_term: Retrognathia; CHPO_term: 下颌后缩

HP:0000400; HPO_term: Macrotia; CHPO_term: 巨耳畸形

HP:0000414; HPO_term: Bulbous nose; CHPO_term: 蒜头鼻

Mapping result:

disorder_id	orpha_number	disease_name	distance	No.1
2823	647	Nijmegen breakage syndrome（综合征如奈梅亨破损综合症）	0.0166	
2418	2636	Microcephalic osteodysplastic primordial dwarfism types I and III（I型和III型小头畸形骨发育不良原始侏儒症）	0.0166	
2739	3047	Blepharophimosis-intellectual disability syndrome, SBBYS type（睑缘病-智力障碍综合征，SBBYS型）	0.0166	
19878	261349	2p15p16.1 microdeletion syndrome（2p15p16.1微缺失综合征）	0.0333	
2439	2671	Neu-Laxova syndrome（Neu-Laxova综合征）	0.0333	

Figure C19-1 Mapping detail of Case19

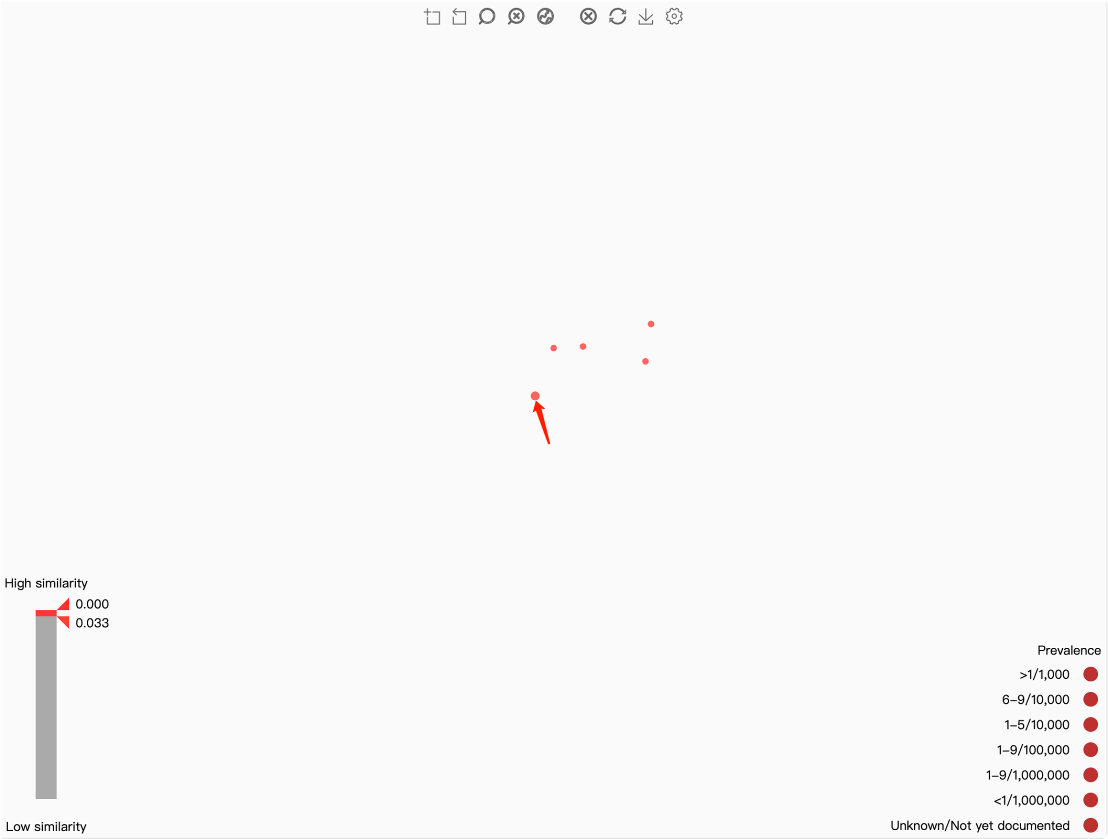

Figure C19-2 The target disease on RDmap of Case19

Case 20:

Publication: Marshall, B.A., Permutt, M.A., Paciorkowski, A.R. et al. Phenotypic characteristics of early Wolfram syndrome. Orphanet J Rare Dis 8, 64 (2013).

Case url: https://ojrd.biomedcentral.com/articles/10.1186/1750-1172-8-64

Diagnosis results: Wolfram syndrome

Case presentation:

Subject	Age	Gender	DM onset	DI onset	Optic atrophy onset	Hearing loss	Diagnosed with WFS	FH WFS	FH DM*	FH hearing loss*	Other problems
WU.WOLF-01	13.1	M	3.5	9	5	9	N/A	N	Father	N	GERD, constipation, headaches, restless legs, hypothyroidism, hypogonadism
WU.WOLF-02	10.9	F	6	7.5	9	N	9.5	N	Mgaunt	Pgf	None
WU.WOLF-03	17.9	M	5.0	6	6	6	6	N	Mgf	mgm	GERD; restless legs; occasional myoclonus

Abbreviations: DI: diabetes insipidus; DM: diabetes mellitus; FH: family history; FTT: failure to thrive; GERD: gastroesophageal reflux disease; maunt: maternal aunt; mcousin: maternal cousin; mgaunt: maternal great aunt; mgf: maternal grandfather; mggf: maternal great grandfather; mggm:maternal great grandmother; mgm: maternal grandmother; mguncle: maternal great uncle; muncle: maternal uncle; OCD: obsessive compulsive disorder; paunt: paternal aunt; pgaunt: paternal great aunt; pgf: paternal grandfather; pgm: paternal grandmother; pguncle: paternal great uncle; puncle: paternal uncle; WFS: Wolfram Syndrome.

Test result:

Entered HPO:

HP:0000819; HPO_term: Diabetes mellitus; CHPO_term: 糖尿病

HP:0000648; HPO_term: Optic atrophy; CHPO_term: 视神经萎缩

HP:0000873; HPO_term: Diabetes insipidus; CHPO_term: 尿崩症

HP:0000365; HPO_term: Hearing impairment; CHPO_term: 听力障碍

HP:0002020; HPO_term: Gastroesophageal reflux; CHPO_term: 胃食管反流

Mapping result:

disorder_id	orpha_number	disease_name	distance
45	95	Friedreich ataxia（弗里德赖希共济失调）	0.0333
812	3463	Wolfram syndrome（Wolfram综合征）	0.0333
110	138	CHARGE syndrome（CHARGE综合征）	0.0333
876	397	Giant cell arteritis（巨细胞动脉炎）	0.0416
94	324	Fabry disease（法布里病）	0.0499
301	2162	Holoprosencephaly（前脑无裂畸形）	0.0499
12856	97229	Riboflavin transporter deficiency（核黄素转运蛋白缺乏症）	0.0583

No.1

Figure C20-1 Mapping detail of Case20

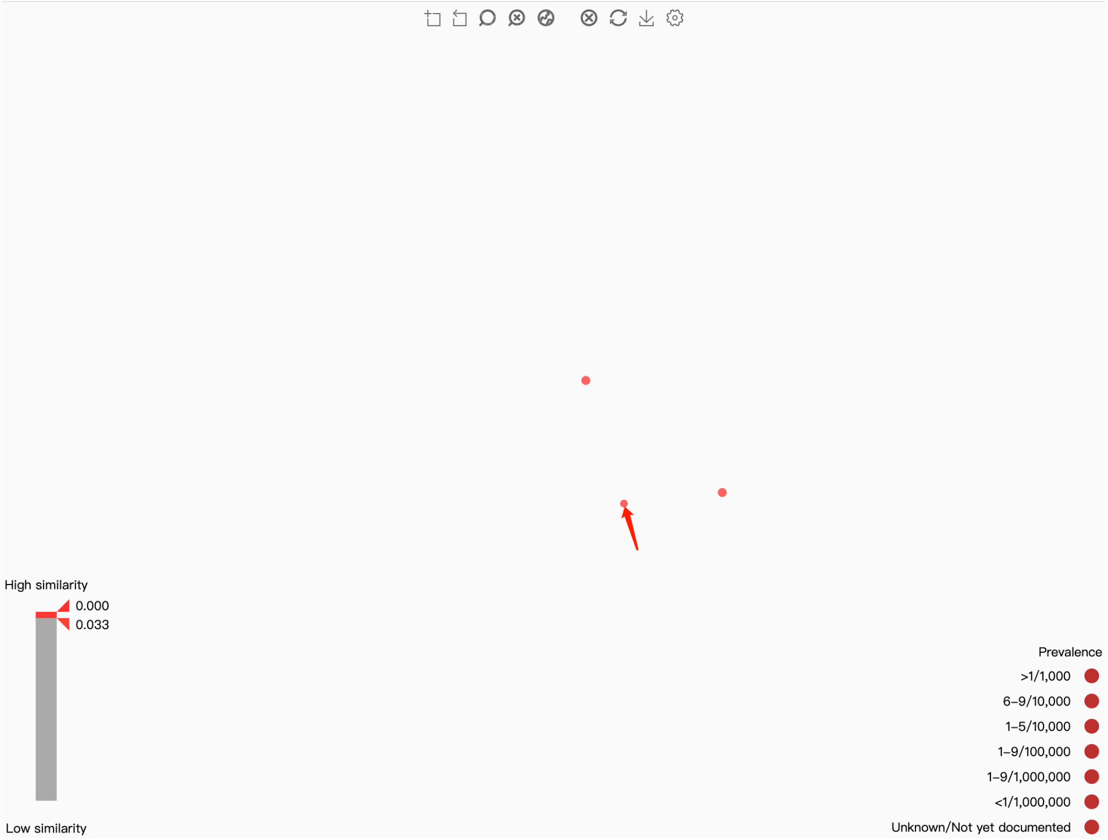

Figure C20-2 The target disease on RDmap of Case20
